# Cushing’s Disease

**DOI:** 10.3390/jcm8111951

**Published:** 2019-11-12

**Authors:** Hiroshi Nishioka, Shozo Yamada

**Affiliations:** 1 Department of Hypothalamic and Pituitary surgery, Toranomon Hospital, Tokyo 1058470, Japan; ayamone@iclud.com; 2 Hypothalamic and Pituitary Center, Moriyama Neurological Center Hospital, Tokyo 1340081, Japan; 3 Okinaka Memorial Institute for Medical Research, Tokyo 1058470, Japan

**Keywords:** aggressive pituitary tumor, Cushing’s disease, Cushing’s syndrome, pasireotide, pituitary carcinoma, pituitary corticotroph tumor, temozolomide, transsphenoidal surgery, USP-8

## Abstract

In patients with Cushing’s disease (CD), prompt diagnosis and treatment are essential for favorable long-term outcomes, although this remains a challenging task. The differential diagnosis of CD is still difficult in some patients, even with an organized stepwise diagnostic approach. Moreover, despite the use of high-resolution magnetic resonance imaging (MRI) combined with advanced fine sequences, some tumors remain invisible. Surgery, using various surgical approaches for safe maximum tumor removal, still remains the first-line treatment for most patients with CD. Persistent or recurrent CD after unsuccessful surgery requires further treatment, including repeat surgery, medical therapy, radiotherapy, or sometimes, bilateral adrenalectomy. These treatments have their own advantages and disadvantages. However, the most important thing is that this complex disease should be managed by a multidisciplinary team with collaborating experts. In addition, a personalized and individual-based approach is paramount to achieve high success rates while minimizing the occurrence of adverse events and improving the patients’ quality of life. Finally, the recent new insights into the pathophysiology of CD at the molecular level are highly anticipated to lead to the introduction of more accurate diagnostic tests and efficacious therapies for this devastating disease in the near future.

## 1. Introduction

In 1932, Harvey W. Cushing reported 12 patients with a serious metabolic disorder, which he ascribed to pituitary basophilic tumors [1]. At present, the term Cushing’s syndrome (CS) refers to the clinical symptoms and signs of inappropriately elevated levels of plasma glucocorticoids. Exogenous (iatrogenic) CS is common, whereas endogenous causes of CS are rare and can be broadly divided into adrenocorticotropic hormone (ACTH)-dependent (approximately 80%; Cushing’s disease (CD), ectopic ACTH secreting tumors, corticotropin-releasing hormone (CRH)-secreting tumors) and ACTH-independent (20%; adrenal tumor, carcinoma, and macronodular hyperplasia (AIMAH)). CD refers to a rare disorder caused by pituitary corticotroph tumors and represents the most common cause (nearly 70% in adults) of endogenous CS. The pituitary tumor overproduces ACTH and lacks hypothalamic-pituitary-adrenal (HPA) axis feedback regulation; thus, excessive amounts of cortisol are secreted from the adrenal glands. Prolonged systemic exposure to elevated cortisol levels results in a significant clinical burden in patients with CD due to comorbidities, increased mortality, and impaired health-related quality of life (HRQoL). Recent studies have clearly demonstrated the impact of early accurate diagnosis and treatment on long-term outcomes [2,3,4,5,6,7]. However, despite significant progress, the diagnosis and treatment of CD remains challenging, and comprehensive endocrine management is often required.

## 2. Epidemiology

Although detailed epidemiologic data on CD is limited, the prevalence is estimated to be nearly 40 per million, and the incidence ranges from 1.2 to 2.4 per million per year, according to several population-based studies [8,9,10]. The prevalence may be underestimated due to unrecognized patients with mild and/or atypical symptoms and patients with a cyclic form of CD [2,11].

Among adults, CD affects women 3-times more frequently than men, and symptoms commonly appear between the third and sixth decade of life. It has been reported that CD appears at a younger age and with a more severe clinical presentation in men than in women [12]. In older adults, a lack of difference in the prevalence of CD between men and women has been reported.

Approximately 75–90% of CS cases in children are due to CD. CD is uncommon in children under 6 years of age; adrenal causes of CS are the typical etiological factors in younger children [13,14]. As in adult patients, there is an overall female-to-male preponderance in children and adolescents with CS, which decreases with younger age [14].

Recently, Wengander et al. [15] suggested that the proportion of CS patients with ectopic ACTH syndrome is higher than that reported previously. Among the CS cases studied, approximately half were caused by CD, one-fourth by ectopic ACTH-secreting tumors, and one-fourth by adrenal disease. In contrast, Hirsch et al. [16] reported that the relative proportion of adrenal causes of CS is rising, probably because of the increasing detection of cortisol-secreting adrenal incidentalomas associated with milder hypercortisolism.

## 3. Molecular Pathophysiology

### 3.1. Genetic Susceptibility

Most pituitary corticotroph tumors causing CD develop sporadically, and only a few cases involving various hereditary endocrine syndromes [17,18,19,20]. These include familial isolated pituitary tumor (FIPA; *AIP*), multiple endocrine neoplasia type 1 (*MEN1*) and type 4 (*CDKN1B*), Carney complex (*PRKAR1A*), and DICER 1 syndrome (*DICER1*). Corticotroph tumors account for about 5% of all pituitary tumors in FIPAs. To date, no germline mutations in *GNAS* or *PRKAR1A* have been reported in CD. Germline *DICER1* mutations have been described in pituitary blastoma, a rare cause of infantile-onset CD [21,22].

### 3.2. Genetic Profile

The pathogenic mechanisms of corticotroph tumors remain largely unknown [23]. One of the recent important advancements is the detection of ubiquitin-specific peptidase 8 (*USP8*) gene mutations in approximately 23–60% of functioning corticotroph tumors [17,18,20,24,25,26,27,28,29,30] (Table 1). The somatic mutations are specific for corticotroph tumors and lead to the increased EGFR expression and activation of proopiomelanocortin (*POMC*) gene transcription. The phenotype of typical *USP8* mutations represents a CD with small tumors in middle-aged women [19,25,26]. The mutation is infrequent in Crooke cell tumor [25], a histological subtype of corticotroph tumor, which often shows an aggressive clinical behavior [19,31]. Corticotroph tumors with *USP8* mutations have significantly higher expression levels of SSTR5 and MGMT than those with the wild type [25]. Consequently, patients with *USP8* mutant tumors may have better surgical outcomes [25,28,29] and may respond more favorably to SSTR5-targeting somatostatin analogues than patients with wild-type tumors. However, a recent study reported that recurrence occurred earlier and more frequently after surgery in patients with *USP8* mutant tumors [32]. Faucz et al. [33] found somatic USP8 mutations in almost one-third (31%) of tumors from pediatric patients with CD. Pediatric patients with USP8 mutations had more severe overall disease, with higher failure rates of primary surgical resection and an increased risk of recurrence.

Recently it was reported that retrospective review of CD series associated with *USP8* mutation show heterogeneity in biochemical findings and surgical outcomes among the series [28]. Wanichi et al. [28] proposed that further multicenter prospective studies would provide more consistent information about the influence of the corticotroph tumors on the phenotype, responses to treatment and outcome of these CD patients. 

Sesta et al. [34] have shown that corticotroph tumors with *USP8* mutation present a more “typical” corticotrope phenotype and reduced expression of several genes associated with protein degradation. On the other hand, *USP8* mutations occur in functioning and nonfunctioning corticotroph tumors. Bujko et al. [30] demonstrated that *USP8* have pleiotropic effect, not limited to EGFR signaling and affect expression levels of many genes involved in different pathways. The protein targets of *USP8*, that could be potential targets for the therapeutic approaches, are still unclear [25,35].

All *USP8* mutations identified in CD were somatic heterozygous single-point mutations, except a recent case report that described a *de novo* germline *USP8* mutation in a pediatric patient with CD [36]. In addition to recurrent CD, this patient presented with developmental delay, dysmorphic features, and other manifestations, which may characterize a new genetic syndrome.

Chen et al. [37] reported that frequent mutations of *USP48* and *BRAF* V600E were detected in corticotroph tumors carrying wild-type *USP8*. Recently, Sbiera et al. have shown that *USP48* mutations are relatively frequent in *USP8* wild-type tumors and enhance CRH-induced hormone production in a manner coherent with sonic hedgehog activation [38]. *BRAF* V600E variants were extremely rare in corticotroph tumors while *TP53* pathogenic variants were more frequent than previously assumed, especially in the larger tumors. In contrast, overexpression of HSP90 is involved in CD and causes partial glucocorticoid resistance [20,24,39,40].

## 4. Clinical Features

Approximately 80–90% of the pituitary corticotroph tumors that cause CD are micro-tumors, most of which are very tiny tumors enclosed within the sella turcica [19]. Thus, patients with CD presenting with signs of sellar mass effect, such as visual disturbance, are uncommon; however, these patients do present with various symptoms of hypercortisolism and comorbidities. Moreover, the clinical presentations can be highly variable, and the diagnosis tends to be delayed for about 2–4 years [2,3,5,8,9]. In general, clinical features are less apparent in men than in women [41,42].

### 4.1. Metabolic Syndrome

The main components include visceral obesity, protein-wasting symptoms, hyperglycemia, and dyslipidemia. Multiple metabolic morbidities persist at least partially, even after remission of CD.

#### 4.1.1. Visceral Obesity

Centripetal (visceral) fat deposition is the most common and often the initial symptom in patients with CD [41,42]. Abnormal fat distribution tends to develop in the face (moon face) and the dorsocervical (buffalo hump) and supraclavicular fat pads. This specific fat distribution often resolves after normalization of cortisol levels and is an important finding for distinguishing CD from simple obesity. Weight gain is a common, but not definitive, feature in patients with CD.

#### 4.1.2. Protein-Wasting Symptoms

Other key clinical findings of CD that are absent in simple obesity include skin thinning, wide purple striae, and proximal muscle wasting, due to the protein wasting effect of cortisol [6]. Skin becomes fragile to minor trauma, leading to frequent bruising, ulcerations, and infections. Muscle wasting, especially in the lower limbs leads to atrophy, fatigue, weakness, and gait disturbance.

Bone wasting caused by hypercortisolism leads to general osteoporosis. Trabecular bone, particularly the vertebral body, is the most frequently affected part, and spinal compression fractures are observed in around half of patients with CD. The prevalence of vertebral and rib fractures may be higher in men than in women [2]. Osteoporosis is more prevalent in adrenal CS than in CD [43]. After sufficient treatment and cortisol normalization, recovery of the bone impairment occurs slowly (6–9 years) and partially [2,5,44].

#### 4.1.3. Hyperglycemia

Impairment of glucose tolerance is a well-known comorbidity of CD. Chronic excessive cortisol secretion leads to muscle, liver, and adipocyte insulin resistance. Nearly half of patients with CD are affected by diabetes mellitus [45]. Moreover, appropriate management of diabetes associated with uncontrolled CD is often difficult.

#### 4.1.4. Dyslipidemia

Lipid abnormalities are also a common comorbidity, occurring in nearly half of patients with CD. To reduce the cardiovascular morbidity and mortality, aggressive treatments are necessary for diabetes and dyslipidemia [2,45].

### 4.2. Cardiovascular Disease

Cardiovascular complications represent the leading cause of death in patients with CD. The risk is increased due to the aforementioned comorbidities, including visceral obesity, diabetes mellitus, and dyslipidemia as well as systemic arterial hypertension, atherosclerosis, and thrombosis diathesis [4]. Cardiovascular morbidity risk can persist in most patients after post-surgical remission [2,4,46,47,48,49].

#### 4.2.1. Arterial Atherosclerosis and Hypertension

These are the most common features of CD. Hypertension is observed in more than half of patients with CD and can be difficult to manage without reduction of cortisol levels [4].

#### 4.2.2. Arterial and Venous Thrombosis

Increased susceptibility to thrombosis is caused by lipid and coagulation disturbances. Hypercoagulability can cause crucial thrombotic complications, particularly venous thromboembolism, between 1 week and 2 months after surgery [2,50]. In a systemic review and meta-analysis, the odds ratio of spontaneous venous thromboembolic events (VTE) was 17.82 in comparison with a healthy population [15].

### 4.3. Opportunistic Infections

Infectious complications are characteristic of CD, and consequent sepsis represents one of the most common causes of death. Increased susceptibility to infection is caused by the direct immunosuppression effect of hypercortisolemia [5]. Extremely high cortisol levels are associated with an increased risk of serious infections. The risk of fungal infections depends on the degree of cortisol excess.

### 4.4. Neuropsychiatric Disorders

Psychiatric disorders are extremely common in patients with CD. They are a significant factor for reducing the quality of life and are often difficult to treat without sufficient management of CD [2,22]. The symptoms are highly variable, including depression, irritability, anxiety, and sleep disturbance and do not correlate with the degree of cortisol excess. These psychiatric symptoms significantly improve after remission in most patients but continue in some patients (particularly depression) [2,5,45].

### 4.5. Cognitive Impairments

Short-term memory and cognition are often impaired in patients with CD. Loss of brain volume, particularly hippocampal atrophy, is induced by chronic hypercortisolemia [4]. The structural changes are partially reversible with normalization of cortisol levels, but the resolution of cognitive impairment may be delayed and partial [2,3,5,45].

### 4.6. Other Clinical Features

Systemic edema, particularly in the legs, is a common symptom and is caused by increased permeability rather than congestive heart failure. Renal stones are observed in nearly half of patients [6].

In women, hirsutism and hypogonadism are frequent due to an excess of adrenocortical androgens. Most women experience dysmenorrhea, and infertility is frequent. Loss of libido is also frequent in men.

In children, most patients present with growth retardation. Although visceral obesity and weight gain is also common, the onset of CS is insidious, and the diagnosis tends to be delayed [13,14]. Libuit et al. [51] reported that boys tend to present with higher body mass index (BMI), shorter height, and higher ACTH levels, suggesting a more aggressive form of CD. Decreases in final height and bone mass are observed without sufficient management.

Older patients with CD appear to have a distinct phenotype with a highly catabolic nature, including lower BMI and greater prevalence of muscle wasting [52]. A number of comorbidities tend to be more common in older patients than in younger patients, including hypertension, diabetes mellitus, history of cardiovascular disease, and history of deep vein thrombosis.

Some patients with large and/or invasive tumors may present with signs of a sellar mass effect, such as visual disturbances, double vision, headache, and pituitary insufficiency. In addition, a few patients, including those with Crooke cell tumor (a histological subtype of corticotroph tumor), may develop an aggressive clinical course, including rapid tumor growth and multiple recurrences [19,31]. In very rare occasions, pituitary corticotroph tumors show distant metastasis and/or cerebrospinal fluid dissemination, i.e., malignant transformation.

### 4.7. Impaired Health-Related Quality of Life (HRQoL)

Impairment of HRQoL is significant and characteristic in patients with CD. The clinical symptoms and comorbidities of CD have a clear negative effect on HRQoL, both physically and mentally [5,7,45].

After treatment of CD, the HRQoL partially improves [2,4,5,7,53,54]. In a study involving surgically cured patients of CD, there was a discordance between biochemical and self-assessed disease status and its impact on HRQoL [55]. In a recent review of HRQoL impairments in patients with pituitary tumors, biochemical remission of CD was associated with the smallest improvement in QoL measurements, compared to that observed in other tumors [53]. In patients with remission, shorter duration of remission, female sex, older age, older age at diagnosis, and hypopituitarism were found to negatively influence HRQoL [53]. In a recent large study based on the European Registry on Cushing’s syndrome (ERCUSYN) [54], patients with CD had poorer HRQoL than patients with adrenal cortisol-producing tumors at long-term follow-up. The study also demonstrated that in addition to persistent hypercortisolism, concomitant depression or treatment for depression also played a pivotal role in affecting the patients’ wellbeing, regardless of CS etiology. Meanwhile, CushingQOL, a widely used disease-specific questionnaire for assessing QoL in patients with CS, should be used with caution when used in different settings, as the country of residence appears to have an impact on the interpretation of the questionnaire [56].

## 5. Diagnosis and Differential Diagnoses

In the Endocrine Society’s clinical practice guideline for the diagnosis of CS, testing for CS is recommended for patients with multiple and progressive features of hypercortisolism, in patients with unusual features for age (e.g., osteoporosis, hypertension), in children with a decreasing height percentile and increasing weight, and in patients with adrenal incidentaloma [44,57]. Abnormal fat distribution caused by protein-wasting (i.e., osteoporosis and myopathy) is a highly specific sign of CS [41,42].

Before conducting biochemical testing, a thorough drug taking history to exclude exogeneous glucocorticoid exposure is necessary [58,59]. Glucocorticoids can be prescribed via oral, topical, rectal, and inhaled routes [57].

Since morning plasma cortisol levels fall in normal range in nearly half of patients with CS, they are not useful in distinguishing from normal [42]. As no single test has an ideal diagnostic accuracy for CS, the diagnosis is often challenging and requires a stepwise approach with combinations of appropriate biological and imaging examinations individualized per patient [5,57,60].

### 5.1. Routine Laboratory Analysis

Although there are no routine laboratory investigations specific to CS, each laboratory test may provide some clue with regard to the diagnosis, including findings such as increased neutrophil and decreased lymphocyte and eosinophil counts; hypokalemia; metabolic alkalosis; hyperglycemia; and hypercholesterolemia, and may be useful for the follow-up of treated patients.

### 5.2. Initial Biochemical Tests for the Screening of CS

The guidelines recommend one of the following biochemical tests for initial CS testing on the basis of its suitability for a given patient: 24 h urinary free cortisol (UFC; at least two measurements), late-night salivary cortisol (two measurements), 1-mg overnight dexamethasone suppression test (DST), and longer low-dose DST (2 mg/d for 48 h) [44]. Although these tests are sensitive, simple, and less expensive, caveats for each test must be considered [42,44,57,60,61]. The tests should be performed under controlled conditions, without stressful or pathological conditions that might affect HPA activity. The measured value of serum cortisol may be falsely increased in women with high estrogen levels, such as pregnant women, those taking oral contraceptive pills, etc., because of increased cortisol-binding globulin (CBG) levels [62]. It has also been reported that CD appears at a younger age and with a more severe clinical presentation in men than in women, together with more a pronounced elevation of cortisol and ACTH levels [12].

Data from the ERCUSYN has revealed that there is heterogeneity throughout Europe in the biochemical testing performed to detect hypercortisolism and diagnose CS [63]. The salivary cortisol assay is not frequently performed in the ERCUSYN participating center or in Japan.

#### 5.2.1. Twenty-Four-Hour UFC Level

In general, UFC is considered the best screening test for CS [42,62,64]. UFC measurement provides an integrated assessment of 24 h cortisol secretion, and the UFC level is not affected by CBG concentrations. To achieve the goal of high sensitivity, the guideline recommends using the upper limit of normal for the peculiar assay as the criterion for a positive test [44]. The sensitivity and specificity of 24 h UFC in hospitalized patients is considered to be higher than those of the 1-mg overnight DST [65]. However, it has been reported that UFC is often within the normal range in 9% of CD and 15% of adrenal CS patients [63,66]. Circulating cortisol concentrations are usually normal (or slightly reduced) in obese patients, but severe obesity can raise the UFC [44]. UFC is falsely raised when the volume is greater than 5 L [67] and falsely low when the glomerular filtration rate drops [68].

#### 5.2.2. Late-Night Cortisol (Serum, Salivary)

A random serum cortisol test usually reflects the HPA activity at the time of measurement and is rarely useful for the diagnosis of CS. Loss of circadian rhythm with the absence of a late-night cortisol nadir is a consistent biochemical abnormality in patients with CS [42]. Measurement of late-night serum cortisol levels has a high sensitivity, but the assessment is increasingly replaced by measurement of salivary cortisol levels mainly because of low cost and convenience [42,62,69,70].

Salivary cortisol is a reliable indicator of plasma free cortisol levels unrelated to the saliva production rate and CBG variability [71]. It has a high sensitivity but its specificity may be low, particularly in the elderly as well as in patients with comorbidities such as obesity, hypertension, and diabetes [57,60,72,73,74]. In healthy adults, advanced age has been reported to be associated with higher late-night salivary cortisol levels, with no evident influence of sex or BMI [75]. The proposed reference ranges and cut-off value of salivary cortisol differ immensely because of differences in the assays used [44,72]. It has also been reported that neither normal UFC levels nor normal late-night salivary cortisol levels exclude mild CS [76]. Multiple samples (urine/saliva) and performance of the DST are needed to establish a diagnosis of mild CS.

AACE/ACE stated that late-night salivary cortisol levels are an earlier predictor of recurrence of CD when compared with the UFC level [77]. However, Sandouk et al. [78] recently reported that patients with recurrent or persistent CD may have late-night salivary cortisol levels in the normal range with considerable frequency.

#### 5.2.3. 1-mg Overnight DST

The 1-mg overnight DST is the most popular and convenient suppression test for the screening of CS. A serum cortisol level above 50 nmol/L (1.8 μg/dL) is considered suggestive of CS, with high sensitivity and specificity rates of 80%, respectively. However, nearly 5% of patients with CD have normal suppression, whereas patients with obesity, depression, and chronic illness may lack normal suppression (false-positive) [41,42,65].

In Japan, 0.5 mg overnight DST with a cut-off level of 5.0 μg/dL is used for the screening of CS. Compared with the 1-mg DST, the 0.5 mg DST has a higher sensitivity but lower specificity.

#### 5.2.4. Longer Low-Dose DST (2 mg/d for 48 h)

The standard format of the test is known as the Liddle test [79]. With a cut-off serum cortisol level of 50 nmol/L (1.8 μg/dL), the test has a sensitivity of approximately 90%, but its specificity is not high [62].

#### 5.2.5. Other Examinations

Recent evidence suggests an increased sensitivity and specificity of hair cortisol level measurements in detecting CS [80,81,82]. Its uniqueness is its ability to provide retrospective information about systemic cortisol exposure over months or even years. Hair cortisol levels can be reliably measured in childhood, and the reference range increases with age [83]. Segmental hair cortisol levels may help identify patients with milder forms of CS and/or cyclic CS [80,81,84].

### 5.3. Biochemical Examinations to Diagnose CD

Once the diagnosis of CS is firmly established, the next step is to explore the cause of hypercortisolism. It is most important to differentiate CD from ectopic ACTH syndrome. The differential diagnosis is often challenging and requires combinations of biochemical and imaging studies.

#### 5.3.1. Plasma ACTH Levels

Morning plasma ACTH level does not fully distinguish patients with CD from normal, just like morning plasma cortisol level [42]. In general, measurement of plasma ACTH levels can help differentiate ACTH-dependent CS from ACTH-independent CS. ACTH levels consistently >15 pg/L indicate ACTH-dependent CS. Conversely, when ACTH levels are <5 pg/mL on several occasions, ACTH-dependent CS is more likely [74]. In most patients with CD, morning plasma ACTH levels are in the normal range or are slightly elevated. Patients with ectopic ACTH syndrome tend to have higher ACTH levels than do patients with CD, although there is a wide overlap [41].

#### 5.3.2. High-Dose Dexamethasone Suppression Test (HDDST)

The rationale behind the HDDST is that negative feedback control of ACTH is reset to a higher level in patients with CD than in normal patients. The overnight 8-mg DST is most commonly used. The proposed cut-off point for a positive response is a serum cortisol decrease to 50% or less of the basal level (sensitivity >80%). Although there is no theoretical reason to fix a rigid cut-off value of 50%, the HDDST is mostly negative in patients with the ectopic ACTH syndrome. However, nearly 10% of patients with the ectopic ACTH syndrome show a positive response and some patients with severe CD, particularly those with a large tumor and an extremely high cortisol level, may lack a response [41,42].

#### 5.3.3. CRH Test

The CRH test is used for direct assessment of the pituitary ACTH reserve. Most patients with CD are responsive (ACTH and/or cortisol levels increase by more than 50% and/or 20%, respectively) to this test, whereas patients with the ectopic ACTH syndrome are typically unresponsive. The sensitivity and specificity of the test are 80% and 95%, respectively, using the ACTH response, and 91% and 95%, respectively, using the cortisol response [85]. However, patients with a large tumor and an extremely high cortisol level may lack a response in the CRH test [41,42].

#### 5.3.4. Desmopressin Test

This test induces a positive response (several criteria exists for its interpretation) in approximately 85% of patients with CD. As the V3 receptor is expressed in as many as 30% of ectopic ACTH-secreting tumors [86], its usefulness is limited for differential diagnosis [41,42,74]. It has been reported that this test can be more useful in detecting recurrence after surgery, as normal subjects rarely respond to the test [42,61,87].

#### 5.3.5. Others

Hypokalemic alkalosis is present in most patients with the ectopic ACTH syndrome, whereas it is less common (<10%) in patients with CD.

### 5.4. Imaging Studies to Diagnose CD

#### 5.4.1. Pituitary Magnetic Resonance Imaging (MRI)

**Figure 1:**
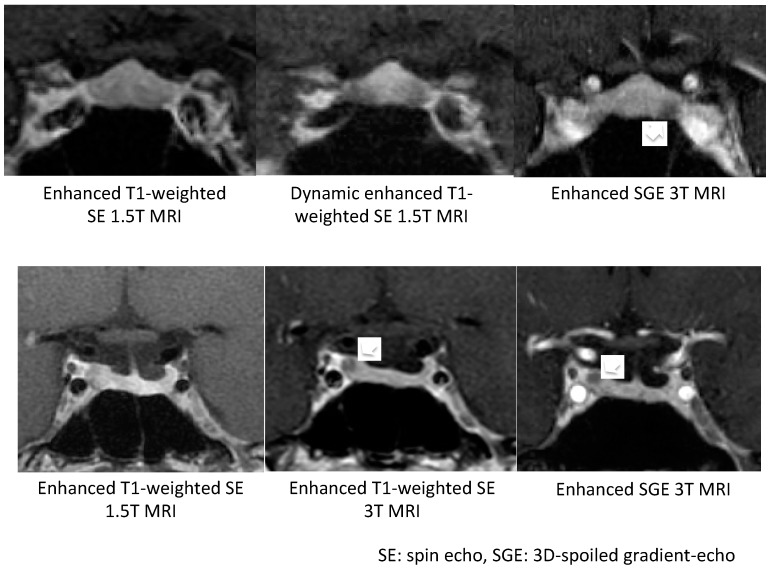
Comparisons of MRI sequences for detecting an adrenocorticotropic hormone (ACTH)-producing micro-tumor (white arrow). The 3D-spoiled gradient-echo (SGE) sequence with 3T-MRI was superior to other conventional MRI sequences in both presented patients.

Micro-tumors can be typically detected on contrast-enhanced MRI images as a less-enhanced mass (the direct finding) and/or an asymmetric convex configuration of the pituitary and the stalk deviation (the indirect finding). With conventional MRI, pituitary micro-tumors can be detected in only 36–63% of patients with CD. One of the most significant recent advancements in the diagnosis of CD is the development of high-resolution MRI and fine techniques (sequences) to detect very small (tiny or minute) corticotrophic tumors [88,89,90]. At present, the 3D-spoiled gradient-echo (SGE) sequence with 3T-MRI, characterized by superior soft-tissue contrast and thinner sections, is significantly superior to conventional MRI sequences, including the dynamic contrast T1-weighted spin echo (SE) sequence, whose spatial resolution is low, for identifying small tumors in CD (Figure 1). It has been repeatedly proposed [89,91,92,93] that the post-contrast SGE-MRI should be used in addition to conventional SE-MRI in the pituitary evaluation of both adult and children with suspected CD. Recently, we reported that the sensitivity and specificity of detecting tumor in 119 patients with CD after the introduction of 3T-MRI were 80% and 100%, respectively [88]. SGE was the best sequence for the detection, and the smallest tumor diameter amenable to definitive diagnosis was 2 mm. The reasons of negative-MRI findings were tumor size, location, and intensity on MRI. Recently, Chatain et al. [94] reported that delayed micro-tumor contrast washout may be detected as FLAIR hyperintensity in otherwise MRI-negative CD cases and proposed adding postcontrast FLAIR sequences to complement SGE. Meanwhile, for patients with gadolinium contraindications, the CISS sequence may be a useful adjunct to T_1_-weighted MRI protocols and an appropriate alternative [95].

**Figure 2:**
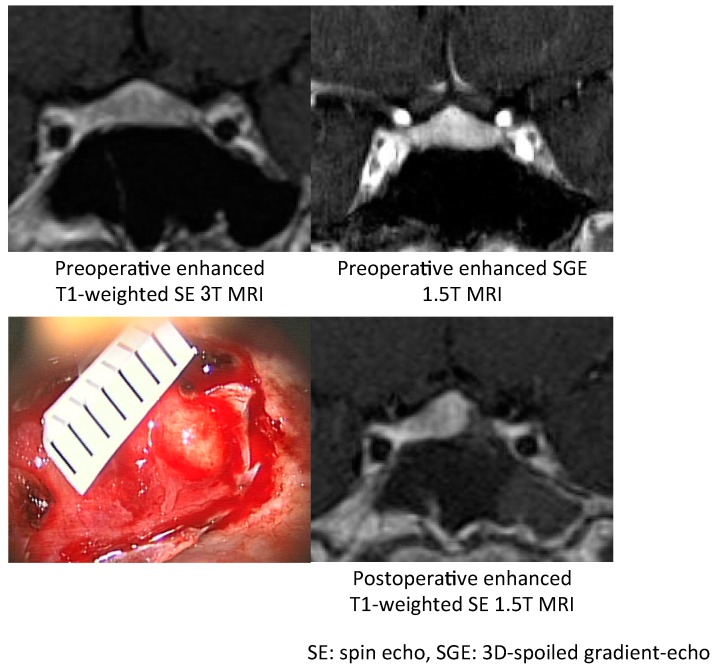
A patient of Cushing’s disease with negative-MRI^(151)^. Extensive MRI studies including 3T MRI failed to demonstrate any findings suggesting tumor but inferior petrosal sinus sampling (IPSS) suggested pituitary origin of ACTH-dependent CD in a 20-year-old woman. A 3-mm diameter micro-tumor was found on the left side of the pituitary as predicted by IPSS. Selective tumorectomy resulted in complete remission of the disease. No abnormal findings were found in preoperative MRI even after review in MRI scans retrospectively comparing them to postoperative MRI findings.

On the other hand, false-positive results may be encountered particularly in cases with pituitary incidentaloma [90], whose prevalence is estimated to be about 10%. It has been proposed that a definitive diagnosis of CD should be established when a tumor discovered on MRI is >6 mm and the biochemical results are compatible [41,74]. Conversely, inferior petrosal sinus sampling (IPSS) is recommended in patients with inconclusive results (Figure 2). However, the use of high-magnetic field MRI and advanced sequences will further improve the sensitivity and specificity of tumor detection, resulting in a decreased necessity of IPSS for the diagnosis of CD [96]. Recently, Law et al. [97] reported that 7T-MRI may preempt IPSS and help in diagnosing standard 1.5T- and 3T-MRI negative CD cases in the future.

#### 5.4.2. Other Imaging Studies

In general, adrenal CT/MRI is unnecessary for patients with ACTH-dependent CS. In patients with CD, hyperplasia may develop in the adrenal gland, and macronodular hyperplasia may develop in up to 15% of cases.

In patients with ACTH-dependent CS and inconclusive results for CD, differentiation from ectopic ACTH syndrome is essential. As the lung is the most frequent site of ectopic ACTH syndrome, a CT scan of the chest is the first step. For the detection of ectopic ACTH-secreting tumors, ^18^F-FDG-PET (fluorodeoxyglucose-positron emission tomography), ^68^Ga-DOTATATE PET/CT, and octreotide (^111^In-pentetreotide) scintigraphy can be beneficial.

^18^F-FDG-PET/CT plays a role in localizing the site of ectopic ACTH-secreting tumors, although it plays a limited role in CD detection. Zhou et al. [98] reported that the serum ACTH level determines the success rate of localization of the primary ACTH-secreting tumor in ^18^F-FDG-PET/CT. Chittboina et al. [21] reported that high-resolution ^18^F-FDG-PET imaging could detect functioning corticotrophic tumors as small as 3 mm. It is more sensitive than SE-MRI but offers no advantages over SGE-MRI. Interestingly, high-resolution ^18^F-FDG-PET-positive tumors had a significantly attenuated response to the CRH test compared with ^18^F-FDG-PET-negative tumors [99].

A recent study demonstrated that ^68^Ga-DOTATATE PET/CT, a high-resolution diagnostic tool for imaging neuroendocrine tumors, is sensitive in detecting primary and metastatic ectopic ACTH-secreting tumors [100].

### 5.5. IPSS

IPSS has been considered the gold standard test for differentiating CD from ectopic ACTH syndrome. As a standard method, serum ACTH levels are measured simultaneously in the catheterized bilateral inferior petrosal sinus before and after CRH administration. A baseline ACTH central to peripheral ratio >2:1 or a CRH stimulated ratio >3:1 is indicative of CD [101]. With regard to baseline vs. CRH-stimulated ACTH gradients, most studies have reported a greater sensitivity of the latter [102]. Similar high-sensitivity results have been reported using desmopressin as an alternative stimulant in IPSS [87,103]. Recently, Chen et al. [104] reported that the sensitivity of IPSS in pediatric CD was low at baseline but increased after desmopressin stimulation. Although its diagnostic sensitivity is considered to be quite high, its diagnostic accuracy is not absolute, and both false-negatives and false-positives occur [92,105]. False-negative results and the absence of an ACTH gradient has been reported in 10–15% of patients harboring a pituitary corticotrophic tumor. In a large series, Wind et al. [106] reported that all patients with false-negative results had peak IPSS ACTH concentrations (before or after CRH) <400 pg/ml. These false-negative results have been attributed to either anatomical (anomalous) variants in the petrosal venous system or technical problems leading to unsuccessful catheterization to the sinus [92,102,107,108,109]. For the latter problem, proper placement of the catheter tips can be routinely confirmed by retrograde venography of the cavernous and inferior petrosal sinuses [92]. To decrease false-negative results caused by the former reason, simultaneous measurement of the prolactin level in the samples has been recommended [109]. In cases with negative IPSS results, the prolactin-adjusted ACTH central to peripheral ratio has been proposed to improve the diagnostic accuracy of CD [59,107]. In a recent review, the results of IPSS successfully confirmed or excluded the diagnosis of CD with 80–100% sensitivity and >95% specificity [102]. Recently, Andereggen et al. [58] reported that asymmetric IPS did not significantly influence the accuracy of ACTH-dependent CS diagnosis with an accurate sampling technique using microcatheters and a clear understanding of the different venous outflow patterns.

The role of IPSS in predicting the localization of micro-tumors remains controversial [76,102,103,106,108,110,111,112,113]. An inter-sinus ACTH ratio >1.4 before or after CRH stimulation has been proposed to indicate the location of tumors with an accuracy of nearly 70% [101]. It has been reported that the prolactin-adjusted inter-sinus ACTH ratio might further improve the accuracy [108,113]. However, De Sousa et al. [110] reported that prolactin cannot act as an independent guide to the diagnosis and lateralization of CD. On the other hand, cavernous sinus sampling (CSS) does not appear to be superior to IPSS for the differential diagnosis and thus its use is currently not routine. However, CSS may increase the accuracy of detecting tumor lateralization in CD [96].

IPSS is the most accurate method to differentiate CD from ectopic ACTH syndrome [47,114], but it is invasive and should be performed in centers at which there are practitioners with a high level of experience. In comparison with MRI (when positive), IPSS was less accurate in predicting intrapituitary tumor location [106]. With improvements in MRI techniques, the number of potential candidates for this invasive method has gradually decreased [96,97]. IPSS must be considered only in patients with definitive ACTH-dependent CS and inconclusive biochemical results and/or negative pituitary MRI [62,106] (Figure 2).

### 5.6. Cyclic CS (Periodic CS)

Cyclic CS is characterized by periods of excess cortisol secretion interspersed with periods of normal cortisol secretion. The cycle durations of cortisol excess vary between several days and years. Although CD appears to be the underlying cause in more than half of the patients with cyclic CS, any etiology of CS can produce the cyclic pattern [11,115]. Jahandideh et al. [116] reported that patients with cyclic CD did not have clear-cut phenotypic differences from those with noncyclic disease. Although cyclic CS is rarely diagnosed, it may be more frequent than generally considered. Alexandrraki et al. [117] reported that cyclicity and variability are not infrequent phenomena in patients with CD with a minimum prevalence of 15%.

Diagnosing cyclic CS can be extremely difficult because of variations in clinical manifestation, the unpredictability of cortisol secretion, and the lack of uniformity in etiology. Any examination can induce misleading results when performed in the trough period [118]. Guidelines recommend the use of 24 h UFC or midnight salivary cortisol levels rather than DSTs in patients suspected of having cyclic CS [44]. Recently, it was reported that the use of late-night salivary cortisol on multiple occasions provided a more sensitive means of detecting cyclic CD than did UFC [116]. Analysis of the segmental hair cortisol level may help identify patients with cyclic CS [80,81,84].

### 5.7. Hypercortisolic States without CS (“Pseudo-Cushing”)

Various physiologic and non-neoplastic activations of the HPA axis can be associated with biochemical and sometimes clinical evidence of endogenous glucocorticoid excess. They can be associated with mild hypercortisolism and may produce test results suggestive of CS, including abnormal dexamethasone suppressibility and mildly elevated UFC [44]. These include depression and other neuropsychiatric diseases, alcoholism and alcohol withdrawal, chronic kidney disease, morbid obesity, poorly controlled diabetes mellitus, glucocorticoid resistance, and pregnancy [42,44]. In these situations, increased cortisol production is thought to be driven by pituitary ACTH overproduction, secondary to a central nervous system disorder or an appropriate adaptive reaction [42]. This functional hypercortisolic state (previously called “pseudo-Cushing”) is usually mild and transient and regresses with the regression of its cause. Alwani et al. [119] have reported that the dexamethasone-CRH test as well as late (mid)-night single measurement of cortisol in serum or saliva demonstrated high diagnostic accuracy in differentiating pseudo CS from true CD.

#### 5.7.1. Depression

Many neuropsychiatric disorders are associated with increases in the HPA axis activity. Patients with severe depression frequently have biochemical hypercortisolism. As there can be many overlaps in clinical features, differentiation between functional hypercortisolism and CS with secondary depression can be challenging [4]. The hypercortisolic state tends to be mild in patients with depression and a positive cortisol response to insulin-induced hypoglycemia, an attenuated ACTH response to the CRH test, and a negative ACTH response to the desmopressin test can be observed, although a wide overlap may exist [42].

#### 5.7.2. Pregnancy

Normal pregnancy is associated with significantly altered glucocorticoid homeostasis. The serum cortisol level is falsely increased because of elevated CBG levels secondary to increased estrogen [62]. Salivary and serum free cortisol levels also increase during pregnancy, particularly in the third trimester [120], by the increase in plasma ACTH levels. However, this does not induce a true hypercortisolic state. A normal circadian pattern of plasma free cortisol and/or salivary and urinary cortisol levels is maintained [42,120]. Guidelines recommend the use of UFC and do not recommend the use of DST in the initial evaluation of pregnant women [44].

## 6. Mortality

Previously, the mortality rate associated with CD was poor; the 5-year mortality rate was 50% [121]. However, current studies have shown that the standardized mortality ratio (SMR) has significantly increased in patients with CD (range: 1.7–4.8) [4,5,8,9,10,44,45,46,48,49,122,123,124,125,126]. According to a large cohort study, there is an increased risk of mortality (hazard ratio [HR] 2.3), VTE (HR 2.6), myocardial infarction (HR 3.7), stroke (HR 2.0), peptic ulcers (HR 2.0), fractures (HR 1.4), and infections (HR 4.9) in patients with CS, even before diagnosis [127]. The mortality risk among patients with adrenal CS (HR 2.4) and CD (HR 2.3) is similar. In general, cardiovascular disease is the most common cause of death, followed by cerebrovascular disease, malignancies, and infectious diseases. In addition, a high rate of suicidal death has been reported recently [111]. The following risk factors contribute to the increased SMR: female gender, older age, persistent hypertension, and impaired glucose metabolism [2].

After treatment, the highest mortality was observed in subgroups of patients with persistent disease, those who were treated with bilateral adrenalectomy, and those who required glucocorticoid replacement [48,123].

The increased mortality risk in patients with CD may persist after “cure” [2,4,46,47,48,49]. According to Clayton et al. [46], the overall mortality risk, particularly due to circulatory disease, in patients with CD who had been in remission for more than 10 years was higher than that in the general population. The total duration of and degree of exposure to hypercortisolism are important factors influencing the persistent elevated mortality [2,46]. Lambert et al. [128] have shown that predictors of mortality in patients with treated CD include duration of exposure to excess glucocorticoids, preoperative ACTH concentrations, and older age at diagnosis. A recent study by Osswald et al. [129] has suggested that the time of exposure to excess glucocorticoids is a better predictor of long-term psychiatric morbidity and QoL than peak serum cortisol at the time of diagnosis. Consequently, for shortening the exposure to active CS, prompt diagnosis and effective treatment is highly desirable [2,3,4,5,6,7].

Among various treatments for achieving remission of CD, pituitary surgery alone has been the preferred treatment to secure an optimal outcome. Clayton et al. [46] have reported that although the overall mortality rate increased in “cured” patients, the overall mortality rate was normal in patients who underwent only one pituitary surgery.

## 7. Treatment

**Figure 3:**
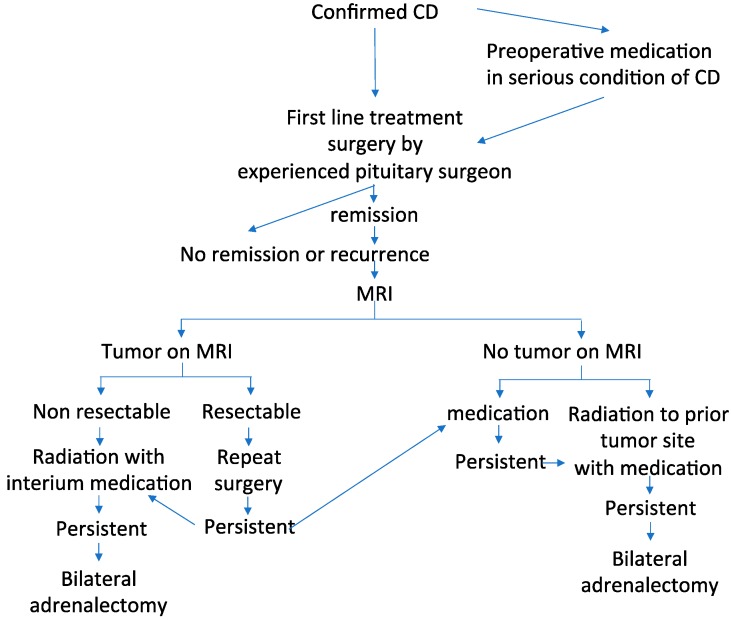
Algorithm of the management of Cushing’s disease. CD: Cushing’s disease.

CD treatment includes (1) normalization of cortisol hypersecretion, (2) reversal of the clinical features associated with CS such as diabetes mellitus, hypertension, muscle atrophy, enhanced cardiovascular risk, depression, memory impairment, and decreased QoL, (3) prevention of or recovery from concomitant comorbidities while restoring life expectancy and QoL to those in the general population, (4) long-term disease control without tumor recurrence, and (5) reversal of optic chiasm compression in cases of pituitary macro-tumor, if present. However, most clinicians find it challenging to treat CD. Hence, multidisciplinary and individualized treatments including surgery, medication, and radiation are needed and their success depends on the expertise of the managing team [4,5,65,130,131]. A proposed algorithm currently used in multimodal management of CD is shown in Figure 3.

### 7.1. Surgical Treatment

Pituitary surgery still remains the first-line treatment for almost all CD patients, and the overall goal is complete resection of the pituitary tumor and correction of hypercortisolism without inducing permanent pituitary deficiencies [5,132,133]. Patients may need preoperative medical treatment to improve hypercortisolism, especially when they are acutely ill due to higher cortisol levels, or surgery will be delayed [131]. It is also recommended that pituitary surgery should be performed by an experienced and skillful surgeon, although neurosurgical techniques have progressively advanced over the last several decades and the accumulated experience has resulted in increasingly improved outcomes [4]. Transsphenoidal surgery (TSS) can be performed by the microscopic or endoscopic approach depending on the neurosurgeon’s preference. In general, neurosurgeons are more familiar with microscopic approach, which provides a binocular stereoscopic view, than the endoscopic approach, which provides a panoramic view. Endoscopic TSS needs a steep learning curve. The data on direct comparison between the two techniques at the same institution are sparse, but the data suggest similar surgical outcomes [134,135]. A recent systematic review and meta-analysis showed that remission or recurrence rates were similar among patients who underwent endoscopic TSS and those who underwent microscopic TSS [136]. However, the most recent meta-analysis reported endoscopic TSS is better than microscopic TSS in CD patients with macro-tumors. The authors concluded that hospitals performing microscopic TSS should consider referring CD patients with macro-tumor to another surgical center performing endoscopic TSS [137]. In contrast, Qiao [132] reported that there was no difference in the remission and recurrence rates between endoscopic TSS and microscopic TSS. Recently, Broersen et al. [138] also reported no clear advantage of endoscopic TSS, describing that three months after microscopic surgery, 74 patients (86%) were in remission. Five-year recurrence-free survival was 89%, and ten-year recurrence free survival was 84% whereas after endoscopic surgery, 39 patients (83%) were in remission and both five-year and ten-year recurrence-free survival were 71%. Finding and consulting an experienced neurosurgeon is a key to biochemical remission without complications [139].

#### 7.1.1. Surgical Approaches for Various Types of Tumors

**Figure 4:**
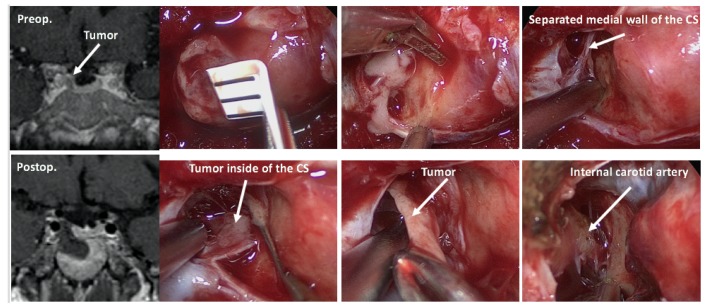
Surgery for a tumor invading cavernous sinus. Fourty-seven-year old female. Small tumor (5 mm in diameter) was located in the right wing of the pituitary invading into cavernous sinus. The tumor was completely removed with separating and excising the medial wall of the cavernous sinus (CS) with the invading tumor inside of the CS Postoperative ACTH and cortisol levels were reduced to <1.5 pg/mL and 1.2 μg/dL, respectively.

**Figure 5:**
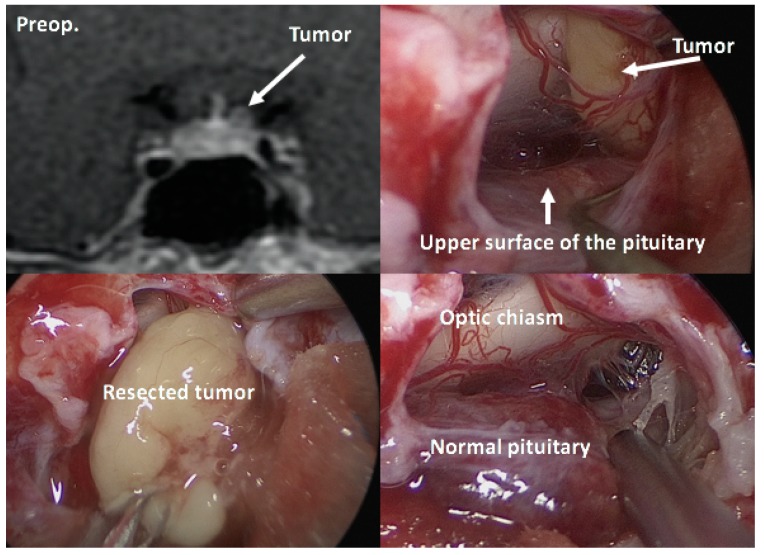
Ectopic pituitary tumor. Thirty-five year-old female. Ectopic micro-tumor (4 mm in maximum diameter) was located in left suprasellar area without any connection with pituitary gland, and the tumor was completely removed via an extended transnasal approach. Postoperative ACTH and cortisol levels are reduced, Table 1 pg/mL and 0.5μg/dL, respectively.

Once the diagnosis of CD has been confirmed, selective tumorectomy via TSS is the treatment of choice for a majority of CD patients, associated with a complete remission rate of 60–90% and <65% for micro-tumors and macro-tumors, respectively [65,140]. The pituitary gland is exposed horizontally to both the cavernous sinuses and vertically to both the intercavernous sinuses. When a tumor is identified during surgery, selective tumorectomy should be achieved with additional removal of normal pituitary around the tumor or extra-pseudocapsular removal should be performed when a tumor has a pseudocapsule [136,141]. Tumors are aggressively attacked and removed as much as possible even when they show cavernous sinus invasion (CSI) by wide opening of the sellar floor to expose the sinus floor [142,143,144]. CSI should be strictly assessed by direct observation of the entire medial wall when the tumor is dissected and excised from the medial wall of the cavernous sinus (Figure 4). It is recommended to use intraoperative monitoring devices (such as microdoppler and eye movement monitoring device) to avoid internal carotid artery injury or reduce the possibility of postoperative eye movement disturbance [145]. In addition, extended TSS, a simultaneous combined transsphenoidal and transcranial approach or transcranial approach followed by TSS or vice versa, should be considered for few complex pituitary tumors. Few pituitary tumor resection must be performed via that transcranial route, but this may be associated with more significant morbidity and mortality than TSS. An extended transplanum–transtuberculum approach, which was introduced by Weiss [140] and has been developed for the surgical treatment of supradiaphragmatic craniopharyngiomas, may offer an alternative to the transcranial route for (1) tumors developing in or around the pituitary stalk; (2) tumors with subfrontal extension, or (3) tumors with a major extrasellar component. An absolute indication is represented by ectopic secreting pituitary stalk/peristalk adenomas (Figure 5), but this approach is associated with higher morbidity than conventional TSS [143,146,147]. In addition, the simultaneous combined approach is effective for the accurate and safe removal by manipulating from both sides (above and below) simultaneously for surgical treatment of few complex (large/giant and multilobulated) pituitary tumors [148]. Surgical strategies for these various types of pituitary tumors are summarized in Table 2.

#### 7.1.2. Surgical Treatment of MRI-Invisible Tumors

**Figure 6:**
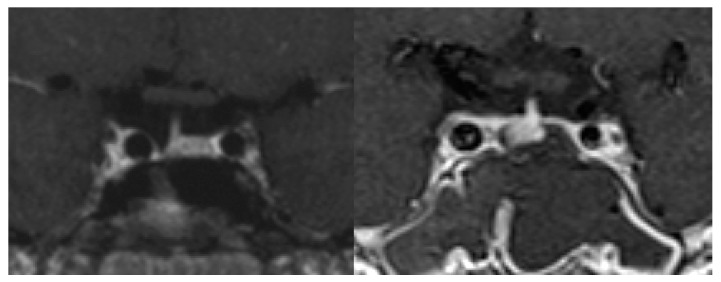
When any reliable tumor is not detected throughout an extensive exploration of the sella, we usually end up hemihypophysectomy of the side suspected by venous sampling results. These two postoperative T1weighted infusion MRIs showing the pituitary after right (**left**) and left (**right**) hemihypophysectomy in cases without any definite tumors during surgery.

Bilateral IPSS with CRH stimulation is the method of choice to confirm CD when any definite tumor is invisible on pituitary MRI. C/P ratio >2 before CRH or >3 after CRH infusion is exceedingly suggestive of CD [76,101]. The frequency of MRI-invisible tumors currently is lower than what has previously been reported (17–63%) [134,149,150]. MRI-invisible tumors accounted for 8.7% patients in a study by Yamada et al. [145], possibly because recent high-field-strength MRI uses various methods including SGE and/or dynamic study with thin sections [88,89,90,145,151]. For the treatment of MRI-invisible tumors, TSS should be considered the first-line treatment if IPSS suggests pituitary tumor. Other therapeutic options must be considered in patients with negative IPSS [151]. In a study, remission after TSS was seen in 28.6% patients; both the MRI and IPSS had revealed negative findings [143]. Therefore, as suggested by other reports [151,152], transsphenoidal exploration should be considered even in MRI-invisible and IPSS-negative tumors if the ectopic source cannot be identified after further detailed body imaging and if the patients agree with surgery after informed consent. The following surgical procedure is recommended: the pituitary gland should be widely exposed horizontally (to both the cavernous sinuses) and vertically (to both the intercavernous sinuses). When a tumor is not visible in surface view after dural opening, the side of the pituitary gland ipsilateral to that suspected by IPSS findings should be first sectioned vertically into three parts (each part: 1.5–2 mm thick) and the presence of tumor should be meticulously searched. When no tumor is seen at this step, the contralateral wing of the pituitary should be sectioned in half, following which the pituitary gland should be cut horizontally to expose the entire anterior and posterior lobes. If no tumor is still identified, bilateral periglandular inspection with visualization of the medial wall of the cavernous sinus and the diaphragm should be performed to search an ectopic micro-tumor located in the periglandular region. When a tumor is identified, selective tumorectomy should be performed and a rim of normal pituitary tissue surrounding the tumor should be removed. When no reliable tumor is detected with this procedure, hemihypophysectomy of the side suspected by IPSS would be finally done (Figure 6). However, total hypophysectomy should be avoided in this situation due to the considerably higher complication rates and unknown postoperative remission rates even after total hypophysectomy [143,153]. Dallapiazza et al. [153] described that in hemihypophysectomy or subtotal hypophysectomy, 30% of the gland is removed from either side and 20% is removed from the inferior aspect; only 20–30% of the normal pituitary gland is left attached to the pituitary stalk. The rate of postoperative pituitary insufficiency is only 15–20% with hemihypophysectomy or subtotal hypophysectomy, which is much lower than total hypophesectomy. Carr et al. [126] also described that failure to find a discrete tumor at the time of surgery occurs in at least 10–15% cases, even at experienced centers. They further showed the possible usefulness of two-third pituitary gland resection in patients with negative surgical exploration. In addition, membranectomy of the sphenoidal sinus to avoid the possibility of an ectopic tumor in the sphenoid sinus should be also performed in patients with negative sellar exploration [154].

It remains controversial whether negative MRI findings affects surgical results; some studies have reported that the remission rates are lower for MRI-negative micro-tumors [135,143,155], whereas some have shown the absence of a correlation between preoperative tumor detection and disease remission [134,136,139,156]. Pivonello et al. [4] reported that the remission rate of patients with preoperative identification of tumor was 52.6–100%, with mean and median remission rates of 79.5% and 80%, respectively. The remission rates of patients without preoperative identification of tumor were 50–95.2%, with mean and median remission rates of 68.2 and 68%, respectively.

### 7.2. Remission Criteria

The remission rates after surgery vary according to the criteria used for each study. Over the years, different centers have used different criteria, as follows: (1) low immediate postoperative serum cortisol [157,158] and ACTH [159], (2) low immediate 24-hour urine free cortisol (UFC) [160], (3) need for glucocorticoid (GC) replacement [160], (4) absence of cortisol/ACTH response to CRH or DDAVP [161,162,163], (5) return of dexamethasone suppression [163,164], and (6) normal circadian rhythm of cortisol or a combination of these criteria [165]. These are appropriate indices of remission in CD. Testing for morning serum cortisol level alone or combined with UFC level is the most commonly used biochemical assay to determine remission [166].

#### 7.2.1. Immediate Postoperative Morning Serum Cortisol Levels

Testing morning serum cortisol levels obtained early in the postoperative period is the most commonly used and reliable marker to evaluate disease activity after surgery. Surgical success is confirmed by subnormal levels of early morning serum cortisol levels within a few days after the surgery. Typically, early morning serum cortisol levels of either <2 μg/dL (<50 nmol/L) or <5 μg/dL are considered to be indicative of remission [4,167,168]. Therefore, this measurement has been widely used in clinical practice. Complete tumor resection leads to adrenal insufficiency due to the prompt cessation of ACTH production by the tumor and the suppression of normal corticotroph cells. In contrast, normal or high postoperative cortisol levels in the first few days suggest the presence of a tumor residual. The cortisol level cutoff for establishing disease remission has not yet been standardized. In particular, some authors consider disease remission to be associated with cortisol levels lower than 1.8–2 μg/dL during the first week after surgery [169,170,171], whereas others consider a value of 3.0 μg/dL [172,173], 5.0 μg/dL [174] and 10 μg/dL [175]. Undetectable cortisol values immediately after surgery predict a more positive outcome, but there are several studies reporting recurrence despite initially achieving undetectable cortisol levels, suggesting that undetectable postoperative cortisol values do not eliminate the possibility of a recurrence [134,176]. Ramm-Pettersen et al. [161] reported that low postoperative serum cortisol nadir predicts the short-term remission, but not long-term remission. Ayale et al. [177] suggested the following follow-up algorithm to enable early diagnosis and treatment of recurrent CD after surgery: (1) patients with a serum cortisol level of <2 µg/dL after 2–3 days of surgery should be monitored semiannually for 3 years and annually thereafter, (2) patients with a serum cortisol level of 2–5 µg/dL after 2–3 days of surgery may experience persistent or subclinical CD and should be evaluated every 2–3 months until biochemical control is achieved, and (3) patients with a postoperative cortisol level of >5 µg/dL may have persistent disease and second-line treatment may be considered. In contrast, Lindsay et al. [163] mentioned that recurrence rates were similar in patients with a serum cortisol level of <2 μg/dL (9.5%) and in those with a serum cortisol level of <5 μg/dL (10.4%).

Normal morning cortisol levels obtained immediately after surgery may not always represent residual tumor, and the delayed decline of cortisol level may be explained by marked adrenal hyperplasia and a somewhat autonomous cortisol production from chronic exposure to elevated ACTH levels during the active phase of the disease or by late necrosis of residual corticotroph adenoma cells caused by surgical manipulation [140,160,168,178]. Alternatively, transient lack of hypocortisolism immediately after the surgery may reflect a nontumoral corticotroph response to surgical stress [168,179]. Moreover, patients who had undergone preoperative metyrapone or ketoconazole treatment to control hypercortisolism could have higher postoperative basal and CRH-stimulated cortisol levels due to early recovery of suppressed nontumorous corticotroph cells and subsequent restoration of the pituitary–adrenal function [161,180]. Valassi et al. [160] suggested that closely following up patients until hormonal parameters stabilize may be the best monitoring strategy to establish a reliable classification of surgical outcomes and avoid unnecessary immediate repeat surgery or other postoperative treatments.

#### 7.2.2. Other Postoperative Parameters for Evaluating Surgical Results

Some studies have indicated that in patients in remission, early postoperative plasma ACTH levels are distinguished [174,181,182,183]. Flitch et al. reported that subnormal (<10 μg/dL) or low normal (<20 μg/dL) postoperative ACTH levels within the first 7 days after surgery were early markers for complete removal and indicators for long-term outcomes in CD [182]. Hameed et al. [174] also reported that early postoperative ACTH levels correlated well with cortisol levels, and the predictive value for remission with cortisol levels of <2 μg/dL and ACTH levels of <5 pg/mL was 100%. Acebes et al. [181] described that plasma ACTH ≤ 7.55 pmol/L distinguished patients in remission from treatment failures with 80% sensitivity and 97.4% specificity, and serum cortisol ≤ 585 nmol/L with 100% sensitivity and 90% specificity. However, the study by Esposito et al. [184] did not confirm any such positive observation. One recent study indicated that ACTH and dehydroepiandrosterone sulfate levels measured at the time of nadir hypocortisolism may carry additional prognostic value. In addition, an ACTH threshold of 20 ng/L clearly distinguished patients with recurrence from those in sustained remission; the serum cortisol levels were similar between all the patients [185]. Moreover, Bansal et al. [62] reported that patients who had plasma ACTH levels of >20 pg/mL had disease recurrences over time despite their serum cortisol levels being <3 μg/dL. In general, accurate determination of early non-remission is of clinical importance because it may selectively identify patients who will benefit from early repeat surgeries [179].

UFC excretion has also been used as a predictor of remission, and there is a trend toward lower rates of recurrence in patients with low UFC levels. Despite the simplicity of urinary sample collection, current recommendations indicate that UFC measurements should only be used when serum cortisol levels are equivocal [186]. In this case, UFC levels of <20 mg/24 h are suggestive of surgical remission; normal UFC levels (range 20–100 mg/24 h) are equivocal and elevated values suggest residual tumor [187].

Night-time salivary cortisol levels have similar advantages as UFC and have been shown to have high sensitivity and specificity in detecting surgical failure and recurrence in CD [71,188]. In addition, Raff [71] suggested that late-night salivary cortisol levels have lower intrapatient variability than UFC, and Amlashi et al. [189] concluded that late-night salivary cortisol levels may accurately establish remission after TSS and identify recurrence more accurately than 24-hour UFC during long-term follow up. However, owing to the variability of the available salivary cortisol assays and lack of a clear cutoff value, testing of late-night salivary cortisol levels has not been sufficiently validated as a useful predictor of long-term recurrence [190].

Several dynamic biochemical testing methods with CRH or desmopressin in the first few weeks following TSS are used to confirm initial remission and to predict long-term recurrence [4,168,187]. In CD, preoperative CRH stimulation results in an ACTH response from the tumor and not from the suppressed normal gland [191]. Complete surgical resection of the pituitary tumor is associated with suppressed CRH response due to long-standing corticotroph cell suppression in the normal gland [192]. Invitti et al. [66] were the first to demonstrate that an increase of >50% plasma ACTH and serum cortisol levels after CRH administration was significantly correlated with the risk of postsurgical recurrence. ACTH and cortisol levels after CRH stimulation were higher in the patients with recurrence, but there are no identifiable basal or stimulated CRH cutoff values that could capture all the recurrences. Results have been highly variable in determining useful criteria for evaluating CRH stimulation data, and there is no established added benefit over static serum cortisol level measurement [163]. However, Alwani et al. [161] found that CRH-stimulated peak cortisol level of ≥21.7 μg/dL predicted a lack of biochemical remission with a negative predictive value of 100%. Multivariate analysis in our study showed that peak cortisol levels of ≥9.4 μg/dL after CRH stimulation and cavernous sinus invasion were significant factors predicting long-term recurrence [143]. Desmopressin stimulation results in a significant increase in both ACTH and cortisol levels in patients with CD or residual ACTH-secreting pituitary tumor after surgery but not in healthy individuals. Colombo et al. [162] concluded that the maintenance or disappearance of the hormonal response to desmopressin test during postoperative follow up may be related to persistent hypercortisolism e or complete removal of tumor tissues. However, there have been reports of postoperative recurrence despite of complete disappearance of the desmopressin response [161,193]. Losa et al. [193] reported that 18 patients considered in remission showed an increase in ACTH levels after desmopressin response and, on the contrary, 4 patients with normal ACTH levels after desmopressin response had persistent hypercortisolism. Moreover, 3 patients with persistent ACTH response to desmopressin experienced CD recurrence in a few years after surgery. Ambrogio et al. [194] demonstrated that a response to desmopressin reappeared in patients who subsequently developed a recurrence of CD, even years prior to hypercortisolism, and concluded that a change in the response pattern to desmopressin is predictive of CD recurrence and may indicate which patients require close follow up. However, Pendharkar et al. [187] described that there is no conclusive evidence that desmopressin stimulation test in the postoperative period provides any predictive advantage over conventional basal serum cortisol level measurements in predicting surgical success and recurrence risk. Recently, Uvelius et al. [195] demonstrated that a 48-hour, 2 mg/day betamethasone suppression test could predict short- and long-term remission with high accuracy.

#### 7.2.3. Early and Long-Term Surgical Results and Predictive Factors Affecting Outcomes

Remission rates vary depending on the location and type of tumor, the neurosurgeon’s expertise, follow-up duration, and the criteria used to define remission [133] (Table 3). Pivonello et al. [4] reviewed 74 studies published between 1976 and 2014 involving 6134 CD patients with a mean follow-up duration of 64.3 months. In their review, they found that the overall initial remission rate ranged from 25% to 100%, with a mean remission rate of 77.8% (median, 78.7%); the recurrence rate ranged from 0% to 65.6%, with a mean recurrence rate of 13.2% (median, 10.6%). The remission rates in micro-tumors ranged from 48.7% to 100% (mean, 82.1%; median, 85.7%), whereas in macro-tumors, they ranged from 30.8% to 100% (mean, 62.3%; median, 64.1%). The recurrence rates in patients with micro-tumors and macro-tumors were 0–36.4% (mean, 11.7%; median, 10.9%) and 0–59% (mean, 18.8%; median, 13.9%), respectively. Many studies have showed that remission and recurrence rates are worse in patients with macro-tumors than in those with micro-tumors. Consistent with this, in the review by Pivonello et al., the remission rate of invasive tumors ranged from 0% to 67% (mean, 41.1%; median, 43%), whereas the recurrence rate ranged from 15% to 36% (mean, 25.3%; median, 25%), clearly demonstrating worse outcomes for tumors invading the cavernous sinuses or surrounding structures. In their recent review that included 87 studies (8113 treatment-naïve CD patients), Abu Dabrh et al. [191] also reported that the overall remission rate was 76% (95% confidence interval [CI], 72–79%) and the recurrence rate was 10% (95% CI, 6–16%) Higher remission rates were observed with smaller tumor sizes (micro-tumor vs. macro-tumor; 0.83 *vs.* 0.63; *p* = 0.001) and with positive ACTH tumor histology (positive vs. negative; 0.74 *vs.* 0.46; *p* < 0.02). None of the other evaluated variables were found to be statistically significant predictors of remission or recurrence. In adults, the remission rate was 71% (95% CI, 64–79%) and the recurrence rate was 13% (95% CI, 8 to 21%). Higher remission rates after surgery were only predicted by smaller tumor size (micro-tumor vs. macro-tumor; 0.89 vs. 0.67; *p* = 0.01). Through multivariate analyses, a study found that cavernous sinus invasion (odds ratio (OR), 13.0), type of surgery (repeat or primary surgery) (OR, 4.0), and tumor size (OR, 2.7) were significant preoperative factors affecting early postoperative remission [143]. The same characteristics (increased tumor size or invasion of the cavernous sinus) have been reported as unfavorable preoperative prognostic factors in other reports [134,160,196]. In children, the remission rate after TSS was 71% (95% CI, 62–82%) and the recurrence rate was 1% (95% CI, 0–32%). Higher remission rates after surgery were observed with smaller tumor size (micro-tumor vs. macro-tumor; 0.83 *vs.* 0.63; *p* = 0.001). The remission rate in children and adolescent CD patients ranged from 44% to 100% (mean, 77.3%; median, 80%), and recurrence rates ranged from 0% to 42.8% (mean, 11.9; median, 6%) [133]. The remission rate in pediatric patients was almost similar to that among adult patients [197]. Lonser et al. [198] showed that identification of a tumor during surgery, presence of an ACTH-producing tumor (as determined using immunohistochemistry), and presence of a noninvasive ACTH tumor were favorable predictors of initial remission in pediatric patients. Moreover, younger age, smaller-size tumor, and the absence of cavernous sinus invasion or other dural invasion were associated with long-term remission. In addition, a minimum morning serum cortisol level of <1 μg/dL after surgery had a positive predictive value of 96% for lasting remission.

#### 7.2.4. Surgical Complications

In general, experienced pituitary neurosurgeons can perform TSS with very low perioperative mortality (0–1.5%) and low complication rates (2–1.5%) [199]. Deaths after pituitary surgery mostly occur due to myocardial infarction [200], pneumonia infection [201], or meningitis [202]. Most complications are minor and transient, but cerebrospinal fluid leak (up to 8%), bleeding or hematomas (up to 6%), epistaxis (up to 6%), venous thromboembolism (up to 4%), and infection (meningitis, up to 3%) may occur [131]. In addition, diabetes insipidus (3–9%) may also occur, although it is generally transient. Moreover, hyponatremia (10–25%) due to the syndrome of inappropriate antidiuretic hormone secretion and anterior hypopituitarism (2–40%) may also occur [203]. Contradictory to this, Lonser et al. [92] reported that the risks associated with surgery for CD (2–10% morbidity, less than 2% mortality) are similar to those associated with surgery for other pituitary tumors and include vision loss, other cranial nerve injury, vascular injury, loss of pituitary function, diabetes insipidus, delayed hemorrhage, and cerebrospinal leakage. Moreover, the risk of these events is lower after surgery for CD than after surgery for larger pituitary tumors, owing to the small size of most tumors in CD. In contrast, other studies have claimed that surgery for CD has a higher rate of complications than does surgery for other pituitary tumors, owing to the greater number of medical complications such as deep vein thrombosis [178,203].

### 7.3. Treatment of CD after Unsuccessful Surgery or Recurrence of CD

A high rate (70–85%) of remission can be achieved with TSS. However, tumors may recur in up to 25% of patients and require further treatment. Moreover, if surgery is unsuccessful, then a second mode of treatment is required, which may include repeat surgery, medical therapy, pituitary irradiation, and/or bilateral adrenalectomy.

#### 7.3.1. Repeat Surgery

**Figure 7:**
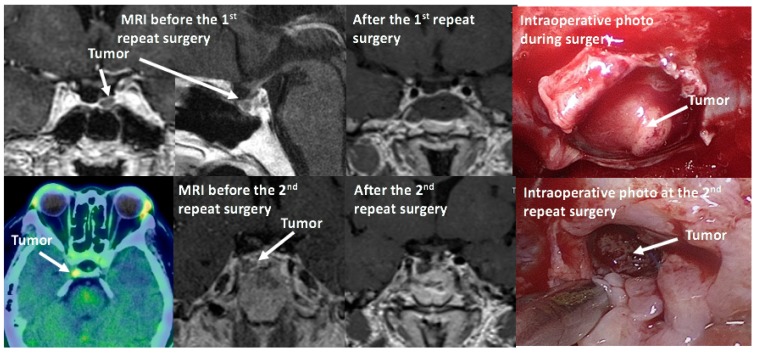
This 52-year-old lady had undergone transsphenoidal surgery for the treatment of pituitary apoplexy (tumor was large macro-tumor at that time). Several years later she showed Cushing’s syndrome and a detailed examination confirmed Cushing’s disease due to pituitary tumor located in the left side of pituitary which was removed by the first repeat surgery. However, both ACTH (91.3 pg/mL) and cortisol level (13.8 μg/dL) were still high. 11C-methionine PET-CT confirmed active small tumor still remained at the right posterosuperior sellar region which had been separated in the primary surgery and was removed by the 2nd repeat surgery (ACTH and cortisol levels reduced to 7.4 pg/mL and 2.6 μg/dL, respectively).

Indeed, the outcomes of repeat surgery for persistent or recurrent CD are poorer and the complication rates are clearly higher than those for primary surgery, even when repeat surgery is performed by a surgeon more experienced than the surgeon performing the first TSS. However, repeat TSS is an effective treatment option for persistent or recurrent CD, and may provide reasonable immediate remission [204]. Rubinstein et al. [200] demonstrated in their review that the mean remission rate after repeat surgery was 54% and 64% in cases of persistent and recurrent CD, respectively. In addition, Valderrabano et al. [205] reported that outcomes after repeat TSS are expected to be modest (immediate remission rate around 50%) with a relapse rate of nearly 50%, implying a long-term remission rate close to 25%. There are many possible explanations for the lower success rate of repeat surgery. Repeat surgery is expected to be more complex technically, mainly due to scar tissue formation and the loss of the anatomical references used by the neurosurgeon [205]. Tumor segmentation may occur during the previous surgery, owing to which some portion of the tumor may be missed during repeat surgery [145]. A detailed comparison of MRI findings before the first operation and before reoperation can help better localize the residual tumor. Recently, Koulouri et al. [206] reported the usefulness of ^11^C-methionine PET co-registered with MRI for detecting residual tumors in cases of persistent acromegaly, which could be differentiated on MRI (Figure 7). Moreover, tumor fibrosis and cavernous sinus invasion are more common in repeat surgery [145,207]. Neurosurgeons tend to perform more extensive surgery during repeat TSS, aiming for total or hemihypophysectomy, especially when no selective tumorectomy is possible after an exploratory approach is used. Remission rates after hypophysectomy are not higher than those observed after selective adenomectomy [134,170,200], but morbidity increases through higher rates of pan- or partial hypopituitarism [202,208]. Valderrabano et al. [209] also observed this association: only 20% of patients undergoing subtotal hypophysectomy achieved remission, in contrast to the 55% who achieved remission after undergoing selective resection.

As suggested by Valderrabano et al. [205], we believe that repeat surgery should only be considered in cases wherein a distinct tumor is visible on MRI, the tumor is in a location accessible during surgery, and there is no cavernous sinus invasion that could limit total excision when hypercortisolism persists or recurs after initial TSS. Most importantly, the repeat surgery should be performed by experienced pituitary neurosurgeons.

#### 7.3.2. Medication

Medical treatment can be considered a valuable alternative to pituitary surgery for CD patients with contraindications to surgery or with CD persistence or recurrence. Medical therapy may also be used preoperatively, especially in patients with severe hypercortisolemia, in order to improve their overall status while preparing for or waiting for surgery. Moreover, medical treatment is also used for patients who have undergone radiotherapy until satisfactory radiation effects were achieved [131]. The effectiveness of medical therapy is usually assessed based on the restoration of normal UFC levels. Medical treatment agents can be categorized as follows: tumor-directed drugs, adrenal steroidogenesis inhibitors, and glucocorticoid receptor antagonists [210]. A combination of drugs might be necessary to achieve eucortisolism [211]. Treatment should be individualized, considering patient characteristics, drug efficacy, and side effects [210].

A. Tumor-Directed drugs

These drugs can target the corticotroph tumor itself and suppress ACTH secretion as well as effectively shrink tumor mass in macroadenomas. Corticotroph tumors often express both somatostatin receptors (SSTRs) and dopamine receptors (DRs). Therefore, pasireotide and cabergoline can inhibit ACTH production by binding to these receptors.

##### Pasireotide

Pasireotide, a multiligand somatostatin analog that can bind to SSTRs, is currently the only pituitary-directed medical therapy approved in the EU, USA, and Japan for the treatment of CD. It has high affinity for SSTR5, the predominant receptor in corticotroph tumors, and the preclinical evidence of a potent anti-ACTH action in corticotroph tumors [212,213]. SSTR activation by pasireotide causes a decrease in cyclic adenosine monophosphate and increase in potassium efflux, resulting in decreased cyclic AMP (cAMP) formation and ACTH secretion. Transduction through signaling pathways involving PTPase, downstream mitogen-activated protein kinase (MAPK), and extracellular signal-regulated kinase 1/2 (ERK 1/2) leads to cell growth arrest or the inhibition of tumorigenesis [214]. Schopohl et al. [215] reported that 50.0% (29/58) and 34.5% (20/58) of patients showed controlled UFC levels (UFC ≤ upper limit of normal) at 12 and 24 months, respectively. The mean percentage decrease in UFC levels was 57.3% (95% CI 40.7–73.9; *n* = 52) and 62.1% (50.8–73.5; *n* = 33) after 12- and 24-month treatment durations, respectively. Recently, Pivonello et al. [216] reported, based on “real-world evidence,” that pasireotide normalizes urinary cortisol levels in at least 68% of CD patients with very mild-to-moderate disease, with consequent improvement in weight, visceral adiposity, and lipid profiles, despite the development or deterioration of diabetes in most cases. This confirms the usefulness of this treatment in patients with milder disease and those without uncontrolled diabetes. Moreover, a recent prospective clinical trial demonstrated that pasireotide was effective for the long-term treatment of CD, especially when surgery fails or is contraindicated [217]. Drug-related adverse events were similar to those of other somatostatin analogues, except for a higher frequency and degree of hyperglycemia [218]. Hyperglycemia was documented in 38.9–81.2% of patients, which may limit this drug’s use in patients with diabetes or glucose intolerance [92,215,216]. Blood glucose levels must be monitored during pasireotide administration, especially in patients with a prior history of diabetes mellitus or impaired fasting blood glucose. The response to pasireotide is usually long lasting, but a few patients with invasive macroadenomas were reported to have stopped responding to this treatment [219]. Pasireotide may also be effective in reducing ACTH levels in Nelson’s syndrome [220]. A long-acting monthly intramuscular drug, pasireotide LAR, is available and has a similar efficacy as the subcutaneously administered pasireotide [221].

##### Cabergoline

Cabergoline, which has a high affinity for dopamine 2 receptors (D2Rs), is a dopamine agonist (DA). A high expression of D2Rs can result in DAs having strong efficacy in reducing tumor size and hormonal secretion. Up to 80% of human corticotroph tumors express functional D2Rs [222]. Pivonello et al. [222] reported that a normalization of cortisol secretion was found in 40% of cases, and all responders showed high D2R expression, whereas all non-responders (except one) did not show high D2R expression. Godbout et al. [223] reported that of 30 patients, 11 showed a complete response (37%) and 4 showed a partial response after treatment with a mean cabergoline dose of 1.5 mg/week. After long-term therapy, 9 patients (30%) continued to show a complete response with a mean cabergoline dose of 2.1 mg/week. In contrast, Ferriere et al. [224], who conducted a multicenter retrospective study of 53 CD patients, showed that about 20–25% of CD patients showed good response to cabergoline therapy, allowing the long-term control of hypercortisolism at relatively low dosages (1.5 mg/week) and with acceptable tolerability. In those responders, metabolic symptoms associated with CD resolved and tumor size decreased or remained stable [225]. However, even in patients who responded initially, subsequent follow-up studies showed no sustained control of hypercortisolism (therapeutic escape), which could result in lower long-term efficacy [223,224]. It also has been suggested that cabergoline can be a safe therapeutic option, especially in patients who are pregnant or desire to get pregnant and thus have limited therapeutic options [226]. Cabergoline is reasonably well tolerated and common side effects include orthostatic hypotension due to the vasodilatory effects of dopamine, nausea, headache, and dizziness. DAs increase the risk of heart valve regurgitation in patients taking much higher doses for Parkinson’s disease [227]. A large cross-sectional study from the UK did not support the clinically concerning association between the use of DAs for the treatment of hyperprolactinemia and cardiac valvulopathy [228]. Doses used for CD (2–3.5 mg/week) are also lower than those used for Parkinson’s disease but higher than those used for prolactinoma; thus, precaution is advised. Gamble et al. [229] recommended that echocardiography screening be restricted to patients at high risk who are taking a high weekly (≥2 mg cabergoline weekly) or cumulative dose of the drug. In addition, the adverse psychiatric effects of DA (impulse control disorders), which are infrequent but potentially have negative effects on patients’ lives, must also be paid attention to [230].

B. Steroidogenesis Inhibitors

Currently available adrenal steroidogenesis inhibitors include metyrapone, ketoconazole, mitotane, and etomidate. In Japan, ketoconazole and etomidate are “off-label” therapies for CD.

##### Ketoconazole

Ketoconazole is an antifungal agent. It inhibits cytochrome P450 enzymes at multiple levels of steroidogenesis, and effectively reduces glucocorticoid and androgen synthesis by inhibiting 11β-hydroxylase and lowers adrenal androgen levels by blocking 17,20-lyase [130]. It can be used as a first-line agent or added to metyrapone therapy. Recently, a French multicenter study (including 14 centers) reviewed data from 200 CD patients with a mean follow-up of 21 months and median ketoconazole dosage of 600 mg/day. They concluded that ketoconazole was highly effective in controlling hypercortisolism; nearly 50% of patients achieved UFC level normalization and an additional 26% showed at least a 50% decrease in UFC levels with a consequent improvement in clinical symptoms (hypertension, hypokalemia, and diabetes mellitus). Nevertheless, 20% of patients were forced to discontinue ketoconazole treatment owing to adverse effects, which were mostly hepatic (mild and severe increases in liver enzyme levels were observed in 14% and 3%, respectively) or gastrointestinal [231]. Liver function disturbance usually occurs during the first week of treatment. Therefore, liver function must be strictly monitored, especially during the first months of treatment [131]. Moreover, male patients may develop gynecomastia, alopecia, and decreased libido owing to antiandrogen action [130,131]. Ketoconazole is administered orally at doses between 400 and 1200 mg, thrice daily. Thereafter, dosage is increased progressively, and the adverse effects are monitored. The onset of action with ketoconazole is slower than that of metyrapone, and this drug requires several weeks to decrease cortisol levels. It demands gastric acidity for absorption, and drugs lowering gastric pH should be avoided. Ketoconazole treatment is effective and has acceptable side effects. However, 7–23% of patients showing an initial response to treatment stop responding to this drug during the later phase of treatment [4].

##### Metyrapone

Metyrapone mostly inhibits 11β-hydroxylase and inhibits 18-hydroxylase to a lesser extent, thereby reducing cortisol and aldosterone synthesis [197]. Metyrapone has been used for diagnosing CS and managing hypercortisolism. Despite the widespread use of metyrapone, data regarding its actual efficacy are scarce, except for one retrospective, multicenter study that included 13 universities in the United Kingdom. In that study, 195 patients with proven CS, including 115 with CD, were included; more than 80% of CS patients (164/195) showed an improvement in cortisol levels, with 55% achieving biochemical eucortisolemia on metyrapone monotherapy [232]. Adverse events, mostly mild gastrointestinal upset and dizziness, occurred in 25% of patients usually within 2 weeks of initiating or increasing the dose; however, the adverse events were reversible [232]. Therapeutic escape is also noted in up to 19% of patients with initial response to treatment [4]. The usual starting dose is 250 mg thrice daily, which may be increased if needed to a maximal dose of 6 g/day four times daily in divided doses. Metyrapone has a relatively rapid onset of action, and the desired cortisol levels are achieved in 75% of patients after 2 weeks of treatment [233]. Metyrapone increases precursor levels and redirects steroidogenesis with mineralocorticoid and androgenic activity; therefore, it may not be the best choice of drug in young women. Increased deoxycorticosterone levels (mineralocorticoid precursors) may cause persistent hypertension, edema, and hypokalemia. Similarly, increased adrenal androgens in women may contribute to persistent/new onset hirsutism and acne [130,140]. However, in practice, the most commonly reported side effect was diffuse gastrointestinal discomfort [232]. Although this medication is relatively well tolerated, higher doses may cause symptoms such as adrenal insufficiency, and thus, appropriate hydrocortisone replacement therapy is needed (block-and-replace regimen). Metyrapone can be used alone or in combination with ketoconazole for the rapid control of hypercortisolism [197].

##### Mitotane

Mitotane is an oral cytotoxic agent mainly used in the management of adrenocortical carcinoma. It is also an inhibitor of multiple enzymes in the adrenal cortex, including cholesterol side chain cleavage enzyme, 3-b-hydroxysteroid dehydrogenase, 11b-hydroxylase, and aldosterone synthase [131]. The starting dose is 500 mg at night, and the dose needs to be titrated up every few weeks to a total dose of 3–4 g/day. Unlike other steroidogenesis inhibitors, mitotane has a slow onset of action, and there is a 4-week delay to obtain maximal efficacy owing to its accumulation in adipose tissues. Mitotane has a very long half-life of 150 days; therefore, the effects of treatment continue for weeks after discontinuing the medication [234]. There is a small difference between efficacy and toxicity levels. The largest studies reported a remission rate of 80%, but mitotane was used together with pituitary irradiation in most cases [235,236]. In contrast, a retrospective study of 67 patients with CD without a prior history of radiotherapy reported sustained normalization of UFC in 72% of patients after 7 months. The mean plasma mitotane concentration at the time of remission was 10.5 ± 8.9 mg/L, with a mean daily dose of 2.6 ± 1.1 g [237]. The main side effects of mitotane are digestive (nausea, vomiting, diarrhea), neurologic (sleepiness, asthenia) and metabolic (hypercholesterolemia) conditions. Mitotane modifies metabolic clearance of steroids with consequent gynecomastia in men and alteration of contraceptive effects of pills [238,239]. Mitotane is also teratogenic; therefore, pregnancy should be avoided for 5 years after discontinuing mitotane [240].

##### Etomidate

Etomidate is an intravenous anesthetic agent that appears to depress CNS function via GABA. Cortisol synthesis is prevented by inhibiting CYP11B1 with 11-beta hydroxylase activity and cytochrome P450scc at high concentrations. Etomidate is an imidazole compound, and intravenous imidazole derivative can be useful when all of the abovementioned medications fail [131]. Etomidate, being a parenteral medication, can block several steps in the cortisol synthesis pathway and may be used as first-line treatment for severe CD with significant biochemical disturbance, sepsis, and other serious complications such as severe psychosis, as well as for preoperative instability [241]. Clinical practice guidelines suggest that etomidate is useful for patients with life-threatening hypercortisolemia who cannot take oral medications [242]. The most critical effect of etomidate is adrenal suppression that occurs approximately 30 min after administering a single dose and may last for approximately 24 h. Etomidate should be administered in an intensive care setting [243].

C. Combination Therapy

Compared with monotherapy, combination therapy is expected to have additive or synergistic effects on the secretion of cortisol and ACTH while reducing drug doses and adverse events [244]. However, there is no valid treatment strategy for all cases, and there are a limited number of studies that have assessed treatment modalities [244]. Medical therapy should be selected according to drug properties and each patient’s particular clinical condition. Combining pasireotide and cabergoline could result in an additive or even synergistic effect. A recent open-label multicenter study showed that an additional one-third of patients (13/39) who started pasireotide and add-on cabergoline treatment had normalized mean UFC after 35 weeks [214]. Pasireotide and/or cabergoline in combination with steroidogenesis inhibitors has also been used. Pasireotide with stepwise addition of cabergoline and ketoconazole when a complete response was not achieved. This stepwise addition of first cabergoline, followed by ketoconazole, to pasireotide increased UFC normalization rates from 29% to 53% to 88% [245]. A combination of cabergoline and ketoconazole targeting both pituitary ACTH and adrenal cortisol synthesis has also been studied. The combination of ketoconazole with cabergoline in 14 CD patients could lead to UFC normalization in 79% of patients, although late night salivary cortisol levels remained higher than normal levels. In contrast, neither drug succeeded in controlling hypercortisolemia nor did the choice of starting treatment (cabergoline vs. ketoconazole) affect outcomes [246]. Vilar et al. [247] demonstrated that the addition of relatively low doses of ketoconazole for patients not achieving a full response to cabergoline resulted in UFC normalization in approximately two-thirds of patients, although among 12 patients, 25% experienced reversal of hypercortisolism with cabergoline monotherapy. The simultaneous administration of mitotane, metyrapone, and ketoconazole for treating 11 severe CD patients led to rapid and sustained decrease in UFC levels [211]. This combination therapy was thus proven to be an effective alternative to avoid adrenalectomy in order to control severe ACTH-dependent CS; however, a high rate of adverse events was also noted, with acute adrenal insufficiency being reported in 36% of patients and elevated liver enzyme levels in up to 82% [211].

D. Other Old Steroidogenesis Inhibitors

Aminoglutethimide is another steroidogenesis inhibitor (an inhibitor of cholesterol side-chain cleavage, 11-beta-hydroxylase, and 18-hydroxylase) that has mostly been replaced by newer agents with better efficacy and lower toxicity. Trilostane (an inhibitor of 17-beta-hydroxysteroid dehydrogenase, 3-beta-hydroxysteroid dehydrogenase, and 17-alpha- hydroxylase/17,20-lyase), which is a weakly active agent, has been used together with metyrapone in Japan.

E. Glucocorticoid Receptor Antagonists

Glucocorticoid receptor antagonism may be a new approach for controlling clinical manifestations of hypercortisolism in CD patients who do not respond to multimodal therapies [248]. Currently, mifepristone is the only available drug with anti-glucocorticoid properties.

##### Mifepristone

Mifepristone blocks the actions of cortisol at the tissue level without a concomitant reduction in serum cortisol levels. It has a rapid onset of action and can be used to treat acute complications of CS, especially cortisol-induced psychosis [249]. The Study of the Efficacy and Safety of Mifepristone in the Treatment of Endogenous Cushing’s Syndrome demonstrated improvement in diabetes and hypertension in 60% and 40% of CS patients, respectively [249]. The study also showed that the overall clinical response, as determined based on data from eight clinical categories, improved in 87% of patients, although ACTH and cortisol levels increased. However, 88% of patients experienced adverse side events such as nausea, headache, fatigue, hypokalemia, arthralgia, and vomiting. ACTH-related long-term effects were analyzed in patients who entered the extension phase of this trial of 43 CD patients treated with mifepristone (300–1200 mg/day). Long-term mifepristone treatment was associated with a ≥2-fold dose-dependent increase in ACTH levels in 72% of patients, and this increase generally remained stable over time. They also noted that corticotroph tumor progression and regression occurred over time. Adrenal insufficiency and hypokalemia have been reported as major severe disadvantages of mifepristone, although they are uncommon [250]. Acute adrenal insufficiency should be carefully examined because it may not be easily reversible by glucocorticoid (dexamethasone) administration. Prolonged administration of glucocorticoid treatment is necessary to overcome the glucocorticoid receptor blocking effect after mifepristone withdrawal [251]. Moreover, increased cortisol levels can lead to severe hypokalemia and/or hypertension owing to excessive cortisol activation of the mineralocorticoid receptor, which responded to spironolactone administration [252]. Mifepristone also has an antiprogestin effect that is often associated with vaginal bleeding owing to endometrial hyperplasia in women [253]. Finally, significant drug-drug interactions exist owing to mifepristone’s effect on a number of cytochrome P450 enzymes [248].

F. Emerging Medical Therapy

Several novel steroidogenesis inhibitors, including levoketoconazole, osilodrostat (LCI699), and ATR-101, are currently in development and are undergoing clinical trial [197,254]. Levoketoconazole is a more potent enantiomer of ketoconazole and is proposed to achieve similar effectiveness with smaller doses and have less pronounced side effects than ketoconazole [197,254]. Osilodrostat is an oral nonsteroidal corticosteroid biosynthesis inhibitor that inhibits 11 beta-hydroxylase and aldosterone synthase with a higher affinity than metyrapone and has a longer half-life. Hypokalemia is usually mild, and no hypertension is observed, despite the increase in the precursor 11-deoxycorticosterone levels. Hirsutism and acne occurred in one-third of women, which was attributed to elevated testosterone levels [254,255]. ATR-101 is a selective and potent inhibitor of acyl-coA acyltransferase 1, inhibiting cholesterol esterification from cholesterol that results in decreasing the substrate supply for steroidogenesis in the adrenal glands. ATR-101 reduces cortisol levels by 50% ± 17% in dogs and is currently being assessed in a phase II, double-blind study in humans [254,256]. Other agents currently under (preclinical) study for the medical control of hypercortisolemia include roscovitine, retinoic acid, and gefitinib. Cyclin-dependent kinase (CDK) 2 and cyclin E normally activate proopiomelanocortin (POMC) transcription and cell proliferation during the corticotroph cell cycle [257]. Roscovitine (R-roscovitine, CYC202, and seliciclib), a 2,6,9-trisubstituted purine analog, can suppress CDK2/cyclin E and inhibit ACTH in animals [258]. Retinoic acid, a vitamin-A-derived metabolite and nuclear receptor ligand, inhibits the transcriptional activity of AP-1 and the orphan receptors Nur77 and Nurr1 in ACTH-secreting tumor cells, resulting in antiproliferative activity and ACTH inhibition in experimental ACTH-secreting tumors in nude mice [259]. Normalization of UFC was achieved in 25%~43% of CD patients [260,261]. Vilar et al. [261] concluded that retinoic acid might be an effective and safe therapy for some CD patients, particularly those with mild hypercortisolism. Epidermal growth factor receptor (EGFR) overexpression leads to upregulated cell proliferation and is associated with many cancers. Blocking EGFR induces reduced POMC promoter activity with antiproliferative activity in a dose-dependent manner [262]. Fukuoka et al. [263] reported that blocking EGFR activity with gefitinib, an EGFR tyrosine kinase inhibitor, attenuated POMC expression, inhibited corticotroph tumor cell proliferation, and induced apoptosis, revealing that inhibiting EGFR signaling may be a novel strategy for treating CD.

Future possible agents that are not yet in clinical trials include heat shock protein 90 inhibitors (Silibinin), testicular orphan receptor 4 inhibitors, histone deacetylase inhibitors, and monoclonal ACTH antibodies [197,214,221,254,262].

These medications are summarized in Table 4.

G. Temozolomide and other potential medications for aggressive or malignant corticotroph tumors

Corticotroph tumors are usually benign, but some of them are aggressive and in rare cases, develop to pituitary carcinomas. Patients with aggressive corticotroph tumors or carcinomas are stupendously difficult to manage with surgery and other treatments. Standard chemotherapeutic drugs such as 5-fluorouracil and carboplatin have disappointing efficacies and do not improve morbidity and mortality rates.

**Figure 8:**
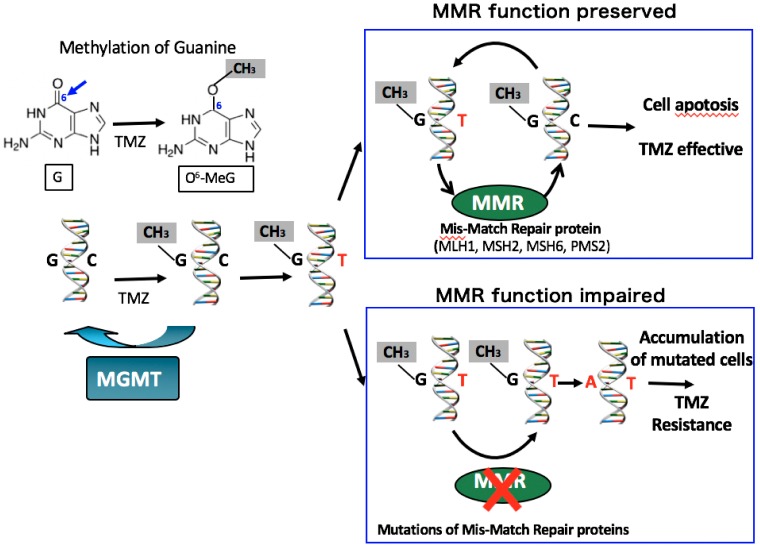
Mechanism capable of expressing or inhibiting an effect of Temozolomide (TMZ). TMZ exerts its cytotoxic effects through methylation at the O6 position of guanine (G) which gives rise to DNA adducts and consequently, tumor cell apoptosis. In contrast, O6-methylguanine-DNA methyltransferase (MGMT) is a DNA repair enzyme that counteracts the effects of TMZ by removing alkylating adducts from DNA. The mismatch repair (MMR) pathway plays important roles in the removal and maintenance of DNA base mismatches caused by incorrect insertions or deletions arising from DNA replication. Base mismatches are detected by the heterodimers of MSH2 and MSH6, which assist another heterodimeric complex of MLH1 and PMS2. Unrepaired TMZ-induced O6-methyl-guanine (MeG) can pair with cytosine (C) or thymidine (T) and the nucleotide pair of O6-MeG/C or O6-MeG/T is detected by the MMR system. Keeping O6-MeG intact, only newly synthesized strands are excised, and this repair cycle will be repeated. With this useless cycle, MMR pathway stimulates DNA damage-induced G2 checkpoint and apoptosis during DNA synthesis. Therefore, the inactivation of MMR is associated with tolerance to the cytotoxic effects of alkylating agents.

**Figure 9:**
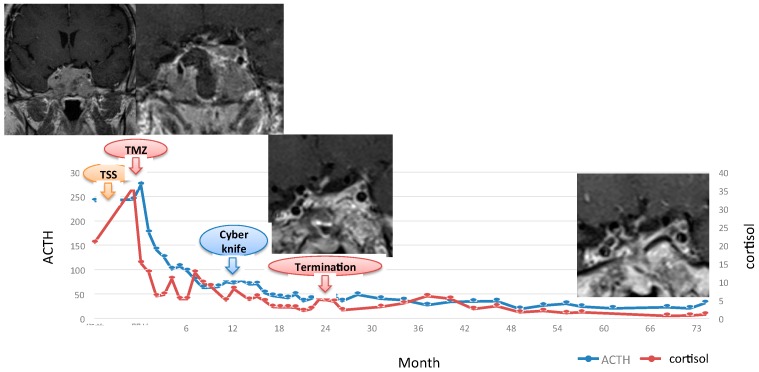
This 33-year-old man was referred to us for seeking repeat surgery with a prior history of one time of transsphenoidal surgery and two craniotomy procedures for the treatment of invasive macro-tumor. Pathology confirmed the tumor was crook’s cell tumor with Ki67 labelling index 4% and O6-methylguanine-DNA methyltransferase was negative. Temozolomide (TMZ) treatment had started after partial removal before radiosurgery because of poor condition of the patient and relatively large residual tumor mass. Not was there a good response from ACTH and cortisol, but the tumor shrunk well to TMZ. TMZ was terminated after 24 cycles and complete remission has continued for 8 years with panhypopituitarism, although cyberknife radiosurgery was performed one year after TMZ start.

Temozolomide (TMZ) is an orally administrated second-generation alkylating chemotherapeutic drug that methylates DNA at the O6 position of guanine, causing a mismatch with thymine during the next DNA replication cycle and leading to cell apoptosis. In contrast, O6-methylguanine-DNA methyltransferase (MGMT) is a DNA repair enzyme that counteracts the effects of TMZ by removing alkylating adducts from DNA. A low expression or the absence of this enzyme strongly correlates with the response to TMZ [264,265]. Furthermore, the preservation of another enzyme system, MSH6 (DNA mismatch repair protein), correlated with the response to TMZ [266] (Figure 8). However, neither drug is sufficiently robust to determine TMZ treatment decisions [267]. TMZ can readily cross the blood-brain barrier, and its action is not cell cycle specific, thus inhibiting all stages of tumor cell growth, even in slow-growing tumors such as pituitary tumors. Since 2006, TMZ, originally approved for use for glioblastoma multiforme, has been used to successfully treat pituitary carcinomas and aggressive pituitary tumors [268] (Figure 9). In 2018, the European Society of Endocrinology Clinical Practice Guidelines recommended the use of TMZ monotherapy as first-line chemotherapy for aggressive pituitary tumors and pituitary carcinomas [269]. TMZ is typically administered in cycles of 5 days every 28 days. The response rate differs according to tumor types and is estimated to be 44% in prolactinomas, 56% in corticotroph tumors, 38% in somatotroph tumors, and only 22% in non-functioning tumors [264]. TMZ is usually well tolerated, but myelosuppression can occur [270].

Potential alternative medications for patients resistant to TMZ include combined everolimus (the mTOR inhibitor) and octreotide therapy in one patient with corticotroph carcinoma, which, however, failed to control tumor growth and ACTH secretion. More clinical cases must be published before any conclusions can be drawn [271]. A novel chemotherapeutic combination regimen of capecitabine and TMZ (CAPTEM) completely regressed or stabilized disease in all 4 patients with aggressive corticotroph tumor [272]. A recent study reported a case of an ACTH-secreting carcinoma that acquired resistance to TMZ and thus was treated with ipilimumab and nivolumab, which suppressed ACTH levels (from 45,550 to 66 pg/mL), and the tumor volume of the dominant liver metastasis reduced by 92% [273].

#### 7.3.3. Definition of Control of Medical Treatments

The definition of clinical or biochemical “control” in a CD patient on medical therapy varies among studies and remains controversial [4,210]. Twenty-four-hour UFC sampling has been commonly used. Furthermore, clinical guidelines have emphasized that despite some caveats, UFC is a good marker to assess response to therapy, except for GC receptor blockers [77]. However, Petersenn et al. [274] analyzed baseline UFC in a large patient cohort with moderate-to-severe CD and clarified intrapatient variability of approximately 50% in 24-h UFC measurements and a lack of clear correlation between UFC and clinical features of CD. Moreover, variability in mean UFC levels increased as UFC levels increased. In contrast, late night salivary cortisol (LNSC) seems to be the most accurate marker compared with UFC, serum cortisol, plasma ACTH, and dexamethasone suppression test in the detection of recurrent CD after surgery [242,275]. Findling et al. [276] suggested that 2–4 measurements of LNSC should be performed on different days to confirm the presence or absence of endogenous hypercortisolism because of its day-to-day variation. Further studies will be needed to determine the optimal method of defining “control” or “recurrence” of CD on medical therapy.

#### 7.3.4. Management and Prevention of Complications

In addition to the treatment of CD itself, the management and prevention of complications associated with CD, such as diabetes mellitus, hypertension, dyslipidemia, osteoporosis, and electrolyte abnormalities, and the prevention and treatment of thromboembolic disease, coronary heart disease, or infection, are extremely important [131].

### 7.4. Radiotherapy

**Figure 10:**
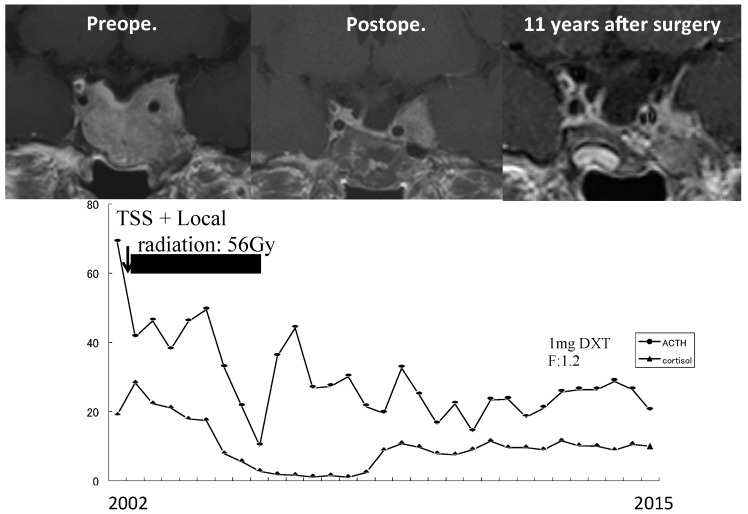
Forty-two-year-old lady with macro-tumor. Local radiation (total 56 gray) was administered after surgery. Her ACTH, cortisol levels have decreased to normal range along with shrinkage of residual tumor mainly located in the outer compartment of cavernous sinus. The patient still continues complete remission 18 years after surgery and radiotherapy.

Radiation treatment (RT) is mainly used when the residual tumor is aggressive and/or invasive and does not respond adequately to surgery and/or medication to control tumor growth and normalize hormonal secretion [210,242,277,278,279] (Figure 10). In general, RT is not indicated in patients in whom the tumor is invisible on MRI [92]. The main disadvantages of radiotherapy are that normalization of ACTH hypersecretion from the tumor may take much time (years) requiring medication until radiation would be successful, and that patients may develop generalized anterior pituitary hormone insufficiency, second intracranial tumor, cerebrovascular disease, cognitive deterioration, or rarely, cranial nerve disturbance [278,280]. However, unlike the medication aimed at only controlling the disease, RT can allow definite cure in patients (radical curative treatment) [278]. Different radiation techniques include fractionated radiotherapy and stereotactic radiosurgery. Conventional external-beam RT (CRT), which is generated from a linear accelerator and delivered in 25–30 fractions to a total dose of 45–50 Gy (typically 2 Gy, 5 times per week). Several technical improvements have recently developed in conventional radiotherapy, such as the intensity-modulated radiotherapy, image-guided radiotherapy and volumetric modulated arc therapy [281]. These techniques allow a more precise dose adjustment, which is useful to spare the vital structures around the target lesion. Castinetti et al. [278] demonstrated that the remission rate after CRT varied between 46% and 84%, although good tumor control was obtained in 93–100% of the cases, and the median time to remission was 24 months. In addition, a high recurrence rate (25–50%) after CRT has been reported [282,283,284]. In contrast, stereotactic radiosurgery (SRS) can be delivered using a Gamma Knife device or a linear accelerator, and proton beam radiation therapy is delivered using a cyclotron [131]. SRS can be administered in a single session or in fractions over several sessions. The principal advantage of the SRS is that it can be delivered in one session compared to CRT, which requires several weeks. The total radiation dose, administered on a single day, is typically 20 Gy. SRS should be reserved for smaller lesions, where the dose to the optic chiasm does not exceed 8 Gy, and the distance from the target and the optic chiasm should be at least 3–5 mm [4,278] For larger lesions or lesions in close proximity to the optic chiasm, fractionated stereotactic radiotherapy using Cyberknife, which is a frameless image-guided stereotactic device, can be used [285,286]. Finally, proton beam radiation can also be used with the theoretical benefit of the lack of diffusion of radiation, which results in a decreased risk of side effects [287]. The efficacy of treatment with SRS seems to be comparable to that of CRT. Tumor control, defined as a decrease in or stability of tumor volume on follow-up MRI after SRS, was achieved in approximately 94% of patients (range, 77–100%), and the initial endocrine remission rate was reported to be 65.8% (range, 26.7–100.0%). The mean time to remission after SRS was 19.9 months (range, 7.5–39.6 months) with no significant differences among the different SRS modalities, while the disease recurred in 20–24% of patients who initially achieved remission [278,279]. A recent international multicenter retrospective study, the largest study published to date, included 278 patients who underwent SRS in a single session, with a mean follow-up of 5.6 years. Cumulative initial control of hypercortisolism was 80% at 10 years. Mean time to cortisol normalization was 14.5 months. Recurrences occurred in 18% of the cases with initial cortisol normalization; adverse radiation effects included hypopituitarism (25%) and cranial neuropathy (3%) [288]. The incidence of remission in CD seems to be similar to the various forms of SRS and CRT, but remission in SRS may occur sooner than that in CRT [92]. Moore et al. [286] reported a time interval of <14 months between surgery, and CK was associated with a significantly greater remission rate; however, the reasons for these observations remain unknown. Some investigators reported a favorable relationship between the discontinuation of steroidogenesis inhibitors immediately before SRS and the achievement of remission [278,289]; however, Mehta et al. [288] did not observe such a relationship. Further studies are needed to understand these intriguing issues.

RT is also used for the prevention or treatment of Nelson’s syndrome potentially occurred 30~50% following bilateral adrenalectomy for uncontrolled CD [290]. In several studies, conventional prophylactic RT achieved pituitary tumor control in 75–100% of the patients with Nelson’s syndrome at a median time of 8 years [291].

### 7.5. Bilateral Adrenalectomy

Bilateral adrenalectomy (BA) has become an important treatment option for refractory CD especially when other treatment options fail. Therefore, BA can be indicated or recommended in patients with severe CD requiring rapid improvement in hypercortisolism when surgery fails to control the disease and medical treatment is intolerant or ineffective [130,292,293]. BA is usually performed using a minimally invasive laparoscopic approach or traditional open adrenalectomy via the anterior or posterior approach. Recently, robotic adrenal surgery has also been applied for BA [294]. When the laparoscopic/robotic technique is applied, postoperative recovery is relatively quicker, with a median hospital stay of 2–3 days and minimized comorbidities [295]. In their review of 549 publications, including 1320 patients, Ritzel et al. [296] demonstrated that 82% of the cases had CD), and the surgical morbidity and mortality of BA were 18% (range, 6–31%) and 3% (range, 0–15%), respectively (surgical mortality was below 1% in CD patients). While residual cortisol secretion due to accessory adrenal tissue or adrenal remnants was observed in 3–34% of the cases, CS relapsed in <2% of the cases. The number of adrenal crisis per 100 patient-years was 9.3. Nelson’s syndrome occurred in 21% (range, 0–47%) of the patients. Mortality was 17% (range, 0–88%) at a follow up of 41 months (range, 14–294 months). The advantage of BA is that it allows immediate control of hypercortisolism; however, life-long glucocorticoid and mineralocorticoid replacement is mandatory after BA. Sarkis et al. [297] recently claimed that BA did not restore QoL that can be expected for CD patients in remission, and they speculated that glucocorticoid substitution plays an important role in this impairment. In addition, Nelson’s syndrome, which involves invasive macro-tumor of the pituitary gland in patients with CD as a consequence of missing glucocorticoid feedback to control tumor cells, should be considered as a severe side effect of BA. Nelson’s syndrome remains a challenging neuroendocrine condition occurring in 8–47% of patients associated with significant morbidity after BA for CD. The primary treatment for Nelson’s syndrome is transsphenoidal surgery. Other therapies, which have been used as adjuvant treatments with surgery, include radiotherapy, radiosurgery, and pharmacotherapy. The most promising pharmacological agents are temozolomide, octreotide, and pasireotide. Prophylactic radiotherapy at the time of BA can prevent Nelson’s syndrome [298]. According to Barber et al. [290], if the residual pituitary lesion is small and/or not visualized on imaging, not prophylactic radiotherapy but follow-up study of annual MRI surveillance, with an annual assessment of ACTH levels, must be recommended. If CD treatments become more refined and shift away from bilateral adrenalectomy, then the incidence of Nelson’s syndrome will naturally decrease. Indeed, Ritzel et al. [66,296] reported 17% of all patients with confirmed CS between 1990 and 2012 were treated with BA, and an Italian multicenter study reported that almost 10% of the CD patients eventually underwent BA, but BA was indicated and performed in only 2 out of 252 patients (0.8%) in our series associated with no case of Nelson’s syndrome [143].

## 8. Pathological Characteristics

### 8.1. Histological Examination

The first step is to confirm whether the submitted tissues are from a normal pituitary gland or a pituitary tumor. Then, immunohistochemical classification based on hormone production by the cells is accomplished, and eventually, prognostic information and treatment options can be determined by means of some biomarkers and by molecular/genetic/epigenetic investigation [299]. The tissue samples obtained during surgery for CD are usually quite small because most tumors in CD are tiny micro-tumors. In contrast, normal pituitary tissues are often submitted to confirm the absence of tumor. Caution must be exercised to prevent misdiagnosis of corticotroph tumor or diffuse corticotroph hyperplasia, especially when normal pituitary tissue shows diffuse ACTH-positive staining. Misdiagnosis may occur when small tissue samples obtained from the central portion of the pituitary, where normal corticotrophs are abundant, or from a portion with basophilic invasion. To avoid this, silver and reticulin stains are significant to demonstrate reticulin fibers. The acinar reticular pattern is conserved in the normal pituitary tissue, while it is lost in tumor tissues; further, it may be enlarged in cases of hyperplasia. Moreover, in 75–80% of patients with chronic hypercortisolism caused by any etiology, the corticotrophs in the normal pituitary undergo changes due to which cytoplasmic granules are replaced with homogenous hyaline material. These changes are known as Crooke’s hyaline changes, and their identification is of paramount importance. The presence of these changes is associated with the degree of hypercortisolism and individual susceptibility to it [300]. These changes involve the massive accumulation of perinuclear cytokeratin under the effect of excessive glucocorticoid levels. This accumulation can be demonstrated using a low molecular weight keratin (CAM5.2) immunostaining, which displays a strong ring-like pattern around the nucleus which displaces PAS and ACTH-positive granules to the cell periphery. Crooke’s hyaline changes are never present in the absence of hypercortisolism. Consequently, their presence can be used to confirm CS in patients in whom no signs of CS are noted during the surgical exploration of the pituitary. Conversely, approximately 20% of the patients with ACTH-positive pituitary tumor at surgery did not exhibit Crooke’s changes, thus, the absence of Crooke’s changes does not always establish the absence of CS [299,300]. Non-neoplastic increase in the number of adenohypophyseal corticotrophs, called corticotroph hyperplasia, in which the reticulin framework is intact but expanded, may be due to the reduced negative feedback effect of glucocorticoids or increased exposure to CRH. Corticotroph hyperplasia may occur in primary hypocorticism (Addison’s disease) or in ectopic CRH producing tumors [301]. It can be focal or diffused and localized to the anterior lobe corticotrophs. Although uncommon, corticotroph hyperplasia causing CD has been described [302,303,304].

Classification of tumors is based on the results of anterior hormone immunohistochemistry. In the absence of distinct hormone content, pituitary transcription factors may be useful to distinguish the differentiation of tumor cells; pituitary specific transcription factor 1 (Pit-1), t-box transcription factor (Tpit), steroidogenic factor (SF-1), estrogen receptor alpha (ER-a), and GATA binding protein 2 (GATA-2) can be employed. Among them, positivity for Tpit is used to confirm corticotroph origin [305]. Corticotroph tumors can be categorized into densely and sparsely granulated types, which refer to the pattern of secretory granules observed by electron microscopy [19,306].

#### 8.1.1. Densely Granulated Corticotroph Tumors

**Figure 11:**
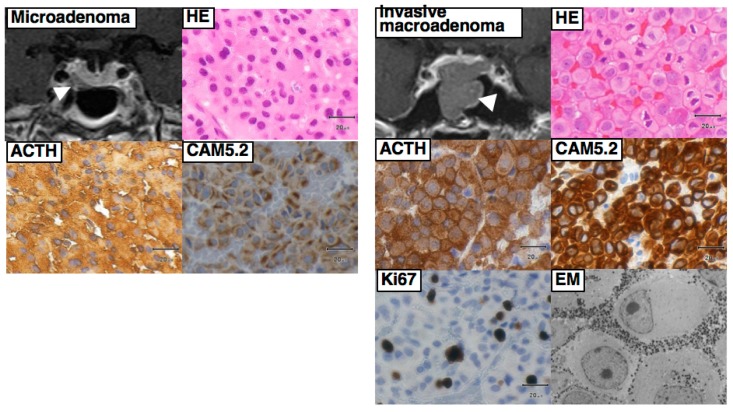
Left two rows showing a typical micro-tumor consisting of densely granulated corticotroph tumor. In contrast, right two rows showing MRI and histology of a Crooke’s cell tumor composed of tumor cells with Crooke’s hyaline change. Ring-like cytokeratin expression is typical with the ACTH expression dislocated to the cell periphery and juxtanuclear region. By electron microscopy, the cytoplasm is filled with intermediate filaments and secretory granules are displaced to the subplasmalemmal area.

They comprise basophilic cells that are diffusely and strongly positive in ACTH and periodic acid-Schiff (PAS) staining [293] (Figure 11). Micro-tumors and small macro-tumors usually have this type of histology. Electron microscopy of the cells shows that the tumor comprises elongate or angular cells with well-developed cytoplasmic organelles. Secretory granules are numerous and show wide variations in size, shape, and electron density; intermediate filaments (type 1 filament) show bundles of perinuclear ring-like keratin filaments, which can be demonstrated by CAM5.2 immunostaining.

#### 8.1.2. Sparsely Granulated Corticotroph Tumors

**Figure 12:**
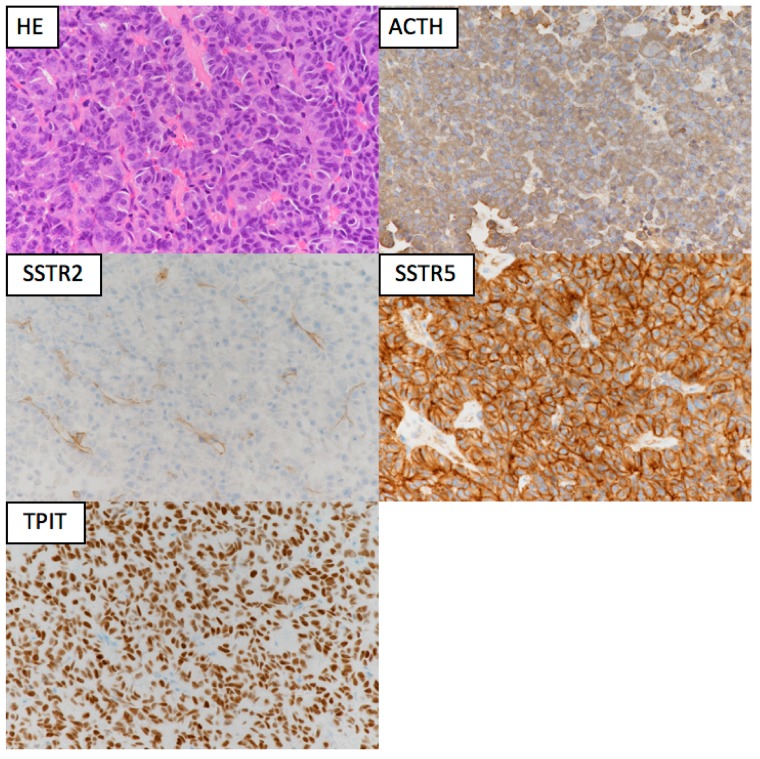
The histology of a micro-tumor showing densely granulated corticotroph tumor. Tumor cells are basophilic in hematoxylin and eosin stains (HE) that are diffusely and strongly positive for ACTH and TPIT. In addition, somatostatin receptor (SSTR) 2 was negative whereas SSTR5 was strongly positive in almost all cells in this case.

Some corticotroph tumors are macro-tumors invading neighboring tissues. In such large, invasive tumors, manifestations of CD are mild or may even be silent, and their histologies show sparsely granulated corticotroph tumors. In the recent epidemiologic study, Mete et al. [302] reported that 52 of 180 corticotroph tumors were sparsely granulated tumors, and that 36 were clinically nonfunctioning and 10 were associated with hormone excess (incomplete clinical data in 6). They are composed of lightly basophilic or chromophobic cells showing only sparse or no PAS and ACTH positivity and are only detected by Tpit (Figure 12). On electron microscopy, the tumor cells display polyhedral cells with poorly developed cytoplasmic organelles, with small and sparse secretory granules. Unlike the densely granulated type, type 1 filament is very few in the cytoplasm.

#### 8.1.3. Crooke’s Cell Tumor

Crooke’s hyaline changes have been considered to occur only in normal pituitary corticotrophs in CD patients, but recent evidence has demonstrated that massive hyaline changes are also observed in the cells of some ACTH-producing tumors. Tumors composed in part (more than 50% of the cells) or entirely of Crooke’s cells are termed “Crooke’s cell tumor” [31]. This type of tumor usually exhibits an aggressive clinical course and is considered to be an aggressive variant of corticotroph tumor. Electron microscopy shows cytoplasm filled with intermediate type 1 filaments and secretory granules that are displaced to the subplasmalemmal area (Figure 11). Although Crooke’s cell tumors are a rare subtype with an estimated prevalence of 4.4–14% in all corticotroph tumors, they are generally aggressive, presenting as invasive macro-tumors and resistant to surgery and radiotherapy with a high recurrence rate [31,307]. Corticotroph carcinomas often arise from preexisting Crooke’s cell tumors [31,308].

### 8.2. Aggressive Pituitary Tumor and Pituitary Carcinoma

Pituitary carcinomas are defined as tumors with craniospinal and/or systemic metastases. Pathological features of pituitary carcinomas are not distinct from those of typical or aggressive tumors, and the only distinguishing feature is the presence of metastases. They are rare, comprising 0.1–0.2% of all pituitary tumors. The majority of carcinomas arise from corticotroph tumors or prolactinomas [309]. The previous WHO classification categorized pituitary tumors as typical tumors, atypical tumors, and pituitary carcinomas. However, the term “atypical tumors” has been deleted in the recent, updated edition of WHO classification because it does not provide an accurate correlation between histopathological findings and clinical behavior [310]. Based on clinical behavior, a pituitary tumor can be considered as aggressive or nonaggressive [311]. Some tumors show features that tend to predict recurrence and resistance to conventional and multimodal therapies. These features include rapid growth, radiological invasion, and a high Ki-67 labelling index. These tumors are referred to as clinically aggressive tumors and must be investigated further and followed up more closely [310]. At present, there is no evidence of a specific genetic or epigenetic background or a common factor that is responsible for the development of pituitary carcinomas [312]. Recent meta-analysis, in which pituitary carcinomas were compared with aggressive tumors, demonstrated that a number of factors related to the stimulation of cell growth, angiogenesis, and invasiveness (cyclin D1, VEGF, MMP9, miR-122, and miR-493) were found to be upregulated, whereas several factors, including growth inhibitors and apoptosis inducers (p16Ink4A, p27Kip1, MT3, BCL-2, Bax, Bcl-X, and MGMT) were downregulated [312,313]. It is speculated that an accumulation of these changes is associated with increased pituitary tumor aggressiveness rather than a carcinoma-specific mutation or epigenetic change that leads to the development of pituitary carcinomas [20]. Trouillas et al. [314] proposed a new clinicopathological classification, graded by invasion and proliferation, and demonstrated its usefulness in a retrospective multicentric, case-control study. In addition, recently, the International Pituitary Pathology Club has proposed a new term, “pituitary neuroendocrine tumor,” which is consistent with that used for other neuroendocrine neoplasms and recognizes the highly variable impact of these tumors on patients because the current classification of neoplasms of the anterior pituitary does not accurately reflect the clinical spectrum of behavior [315].

### 8.3. Information for Postoperative Medical Treatment for CD

The study of expression of SSTR2 and SSTR5, dopamine 2 receptor, or MGMT examined easily by immunohistochemistry can provide useful information when more appropriate medication as adjuvant therapy needs to be chosen [308,316]. The majority of corticotrope tumors (>85%) express SSTR2 and SSTR5 mRNAs and to a lesser extent SSTR1 mRNA (63%) (Figure 12). The membrane density of SSTR subtypes, particularly SSTR2, is affected by hypercortisolism, whereas SSTR5 expression appears to be relatively unaffected by high cortisol levels. Therefore, SSTR5 is predominantly expressed over SSTR2 in patients with active CD [316]. Takeshita et al. [25] claimed that the presence of USP8 mutations may predict favorable responses to pasireotide, which exhibits high affinity for SSTR5. In pituitary tumors, the expression of MGMT but not methylated MGMT promoter can predict the response to temozolomide treatment. Nonmutated aggressive tumors such as Crooke’s cell tumor may respond better to the alkylating agent temozolomide because of their significantly weak expression of MGMT [311].

## 9. Conclusions

A significant burden has been demonstrated in patients with CD due to comorbidities, increased mortality, and impaired HRQoL. Recent evidence indicates that following the long-term exposure to excess glucocorticoids, these burdens persist, at least partially, after CD has been “cured.” Thus, many investigators have emphasized the crucial importance of rapid diagnosis and treatment as well as appropriate follow-up. However, as there is no single test that accurately diagnoses CD, the diagnosis is often challenging and requires a stepwise approach with combinations of appropriate biological and imaging examinations, tailored to each patient’s requirements. The treatment of CD is one of the most challenging tasks in endocrinology. Differentiation of CD from other underlying causes of CS is still difficult in some cases even with an organized, stepwise diagnostic approach. Surgery still remains the first-line treatment for almost all CD patients. Most tumors are micro-tumors in CD, and TSS is therefore an appropriate approach; however, some macro-tumors require extended TSS or a combined, simultaneous TSS and TC approach for safe maximum tumor removal. However, some (approximately 10% of cases) are MRI invisible tumors despite the use of modern MRI methods. Persistent or recurrent CD after unsuccessful surgery needs further treatments including repeat surgery, medical therapy, radiotherapy, or, in few cases, bilateral adrenalectomy. Repeat surgery should only be considered in patients in whom a distinct and accessible tumor is visible on MRI, but it is usually associated with a relatively higher rate of failure and recurrence. Currently available medical therapies include adrenal steroidogenesis inhibitors, tumor-directed drugs, and glucocorticoid receptor antagonists. Adrenal steroidogenesis inhibitors are useful for the short-term control of cortisol excess, but multiple adverse effects and drug interactions often occur. Pituitary tumor-directed drugs have the potential advantage of tumor shrinkage as well as improvement in hypercortisolism. Combination therapy is an alternative strategy to increase treatment efficacy whilst minimizing adverse events. Some novel medical therapies are under clinical trial, and some emerging medical therapies are currently under evaluation. Radiotherapy is an effective second-line treatment especially when surgically inaccessible tumors exist, but the long delay efficacy and side effects associated with radiation must be always considered while adopting radiotherapy. In contrast, bilateral adrenalectomy provides permanent cure of the hypercortisolism, but the chronic adrenal insufficiency and the risk of development of Nelson’s syndrome are always of concern. In general, a multidisciplinary team with collaborating experts is mandatory for the management of this complex disease, and a personalized, individual-based approach is indispensable to achieve the highest success while minimizing the side effects and improving the patients’ QoL. Finally, it is highly anticipated that recent new insights into the pathophysiology of CD at the molecular level will lead to more accurate diagnostic tests and more efficacious therapies for this devastating disease in the near future.

## Figures and Tables

**Table 1:** Reported frequencies of ubiquitin-specific peptidase 8 (*USP8*)-mutation in patients with Cushing’s disease (CD) and their clinical characteristics.

Reincke M (2015) [27]	4/10 (40.0%)	
Ma, Z.Y (2015) [26]	67/108 (62.0%)	smaller tumor size (*p* < 0.001), female sex (*p* = 0.01)
Hayashi K (2016) [25]	21/60 (35.0%)	female sex (*p* = 0.023), smaller tumor size (*p* = 0.0012), lower Knosp grade (*p* = 0.012), higher surgical remission rate (*p* = 0.001)
Faucz FR (2017) [33]	13/42 (31.0%) (pediatric patients)	later age (*p* = 0.03), lower body mass index z-score (*p* = 0.02), higher rate of recurrence or failure to cure (*p* = 0.009)
Albani A (2018) [32]	18/48 (37.5%)	younger age (*p* = 0.028), higher 24h-UFC level (*p* = 0.045), higher recurrence rate (*p* = 0.026), earlier recurrence (*p* = 0.019)
Bujko M (2019) [30]	15/28 (53.5%)	
Losa M (2019) [29]	22/92 (23.9%)	female sex (*p* < 0.05), higher surgical remission rate (*p* = 0.01)
Wanichi IQ (2019) [28]	11/47 (23.7%)	female sex (*p* < 0.00001), lower 24h-UFC level (*p* ≤ 0.017), higher surgical remission rate (*p* < 0.00001)

**Table 2:** Surgical strategies for various types of pituitary tumors.

**Microadenomas or ordinary macroadenomas**
-Conventional transsphenoidal approach
-Removal with adjacent tissue with the help of intraoperative rapid diagnosis, if necessary
-Extracapsular removal, if adenoma has pseudocapsule
-En-bloc excision, if adenoma is elastic hard in consistency
**Tumors with cavernous sinus invasion**
-Aggressive attack to the CS via ethmoid-pterygo-sphenoidal approach with the help of intraoperative monitoring devices
**Multi-lobulated large macro-/giant adenomas**
-Transcranial approach
-Extended transnasal approach
-Simultaneous transcranial and transnasal combined approach

**Table 3:** Literature review of postoperative results after transsphenoidal surgery for Cushing’s disease.

						Remission Rate			Remission Criteria		Recurrence Rate		
	No. of Cases (No. of Female)	Mean Age	Macro	Micro/Invis	Follow-up (Years)	Overall	Macro	m/Invis	F(μg/dL)	Other End Points	Overall	Macro	Micro/Invis
Hameed et al. 2013 [169]	52(38)	45	16	36/8	1.4	83%	75%	86%/NA		D+U+S+GR	12%	NA	NA/NA
Hassan-SmithZK 2012 [118]	72(NA)	40	NA	NA/NA	4.6	72%	NA	NA/NA	<1.8		11%	NA	NA/NA
Honegger et al. 2012 (229)	83	46	11	72/9	3.2	84%	64%	88/78%		D+U+C	7%	0	7%/NA
Ciric et al. 2012 (230)	136(109)	41(20–70)	13	123/23	5.7	83%	31%	90/93%	<5	GR+C	NA	NA	NA/10%
Lambert et al. 2013 [122]	256(NA)	40	NA	NA/NA	6.3	89%	NA	NA/NA	<5	D+U+GR	21%	NA	NA/NA
Alexandraki et al. 2013 [128]	124()		21	103/NA	15	68%	43%	73%/NA	<1.8	GR+U	24%	33%	23%/NA
Sarker et al. 2015 (228)	64(51)	31(onset)	11	53/8	1.7	80%	56%	86/67%	<5	GR+D	NA	NA	NA/NA
Yamada et al. 2015 [137]	252(215)	43	76	176/22	6	83%	71%	94/43%	<3	MRI+D+GR+C	5%	15%	2.7/0%
Shirvani et al. 2016 (231)	96(73)	31	18	78/12	3.7	95%	NA	NA/NA	<5	U	22%	NA	NA/NA
Chandler et al. 2016 [168]	275(215)	40	35	240/103	6.7	80%	66%	89/71%	<3 or N	U	17%	NA	NA/NA
Keskin et al. 2017 (225)	82(63)	44	19	63/13	7.5	72%	90%	68/62%	<2	D+U+S	27%	5%	17/22%
Johnston et al. 2017 [151]	101(73)	47	27	74/25	4.3	82%	63%	89/84%	≤3	D+U+S	7%	12%	6/5%
Brichard et al. 2018 [173]	71(57)	43	13	58/17	6.9	83%	92%	85/71%	<5	D+U+S+GR	18%	25%	14/21%
Saini et al. 2018 (227)	60(45)	25	18	42/8	3.3	72%	NA	NA/NA	<5	D	18%	NA	NA/NA
Feng et al. 2018 (226)	289(NA)	36	28	261/NA	Range 1–3	79%	79%	79%/NA	<5		2%	NA	NA/NA

This Table is an adaptation from Brichard C et al. [173]. Micro-tumor (<10 mm maximum diameter) includes invis adenoma (MRI invisible adenoma), F: morning serum cortisol, D: overnight dexamethasone suppression test, U: 24-h free cortisol, S: midnight salivery cortisol, C: clinical remission, N: normal range, NA: Data were not available.

**Table 4:** Summary of medical therapies for Cushing’s disease.

Medication	Mechanism of Action	Dose (route)	Normalization of UFC (%)	Main Side Effects	Comments
Pituitary tumor directed drugs					
Pasireotide Pasireotide LAR	Binding to mainly SSTR5 resulting in inhibition of hormone secration	600~900 μg 2 times a day (sc) LAR: 10~40 mg/month (im)	30–80%	Hyperglycemia (40~80%), GI and biliary issues, QT prolongation, GH deficincy	Effective in only mildCD, reatment for hyperglycemia or stopping administration (~9%) due to hyperglycemia often required. Combined with cabergoline and/or steroidgehesis inhibitors Chandler
Cabergoline	Binding to Dopaminetype 2 receptor resulting in inhibition of hormone secration	0.5–6 mg/week (oral)	20–40%	GI issues, postural hypotension, headache, cardiac valve sclerosis (high dose), impulse control disorders	Usually well tolerated, short-term response, therapeutic escape often occurs (~25%) and a cause of lower long-term efficacy, off- label use, combined with pasireotide and/or steroidgenesis inhibitors
Roscovitine (seliciclib)	suppressing CDK2/cyclinE	400 mg 2 times a day (oral)	No data	GI issues, hypokalemia	Phase II study
Retinoic acid	Regulation of gene transcription results in reduction of tumor activity	80 mg once a day (oral)	25–40%	Photosensitivity, arthralgias, diarrhea, headache	Phase II study
Gefitinib	Blocking EGFR activity	250 mg once a day (oral)	No data	Intestinal pneumonia, severe diarrhea, dehydration, toxic epidermal necrolysis	Phase II study
Heat shock protein inhibitors (Silibinin)	Downregulation of PTTG1				Future possible agent (preclinical)
Testicular orphan receptor4 inhibitors (MEK-162)	Reducing TR4 expression and inhibiting binding to POMC promoter				Future possible agent (preclinical)
Histone deacetylase inhibitors(vorinostat)	Downregulation of POMC via suppression of the nuclear liver X receptor alpha (LXRα)				Future possible agent (preclinical)
Adrenal steroidogenesis inhibitors					
Ketoconazole	Inhibition of cholesterol side-chain cleavage enzyme, 11β-hydroxylase, 17α-hydroxylase, and 18-hydroxylase	400–1600 mg three-times a day (oral)	~50%	GI issues, abnormal liver function tests (mild 14%, severe 3%), gynecomastia, alopecia and/or decreased libido (in men)	slower onset of action, requiring several weeks to decrease cortisol levels. It demands gastric acidity for absorption. Therapeutic escape occurs in 7 to 23% of patients with initial response
Metyrapone	Inhibition of 11β-hydroxylase and 18-hydroxylase	500–6000 mg two~four-times a day (oral)	75–80%	GI issues, dizziness, hypertension, edema and hypokalemia hirsutism and/or acne (women)	higher doses may cause symptoms of adrenal insufficiency.not the best choice in young women
Mitotane	Inhibition of cholesterol side chain cleavage enzyme, 11β- and 18-hydroxylase and 3β-hydroxysteroid dehydrogenase	1500–6000 mg three times a day (oral)	72–80%	nausea, vomiting, diarrhea, dizziness, neurotoxicity, and dyslipidemia	Permanent adrenal atrophy equivalent to a chemical adrenalectomy. Very long half-life so that it may have effects lasting months after discontinuation. Its use is limited to select cases
Etomidate	Inhibition of cholesterol side-chain cleavage enzyme, 11β-hydroxylase	After initial bolus of 0.03 mg/kg iv, 0.02–0.08 mg/kg per hour	~100% (short period)	Somnolence, myoclonus, vomiting, dystonic reactions, rash, angioedema	Useful for patients with life-threatening hypercortisolemia. Anesthetic agent, and should be administered in an intensive care setting.
Levoketoconazole	Inhibition of cholesterol side-chain cleavage enzyme, 11β-hydroxylase, 17α-hydroxylase, and 18-hydroxylase	400 mg two-times a day (oral)	30–34%	Headache, Back pain, nausea,	More potent enantiomer of ketoconazole Similar effectiveness with smaller doses and less pronounced side effects than ketoconazole Phase III
Osilodrostat (LCI699)	inhibition of 11β- hydroxylase and aldosterone synthase	4–100 mg two-times a day (oral)	79–92%	fatigue, nausea, and diarrhea (mild to moderate) acne and/or hirsutism (women)	It iis similarly to metyrapone, but more potent and with a longer half-life ACTH levels rise significantly and testosterone levels increase in 75% of female patients Phase III
Glucocorticoid receptor antagonists					
Mifepristone	Blockade of glucocorticoid and progesteron receptors	300–1200 mg once a day (oral)	87% (clinical manifestations improvement	Adrenal insufficiency, hypokalaemia and/or hypertention, GI issues, vaginal bleedings, endometrial hyperplasia, dyslipidemiaarthralgia	Prolonged glucocorticoid treatment is necessary Contraindication in women planning pregmancy Cortisol and ACTH levels remain high and cannot be used to titrate therapy
